# Current Advanced Therapies Based on Human Mesenchymal Stem Cells for Skin Diseases

**DOI:** 10.3389/fcell.2021.643125

**Published:** 2021-03-09

**Authors:** Álvaro Sierra-Sánchez, Trinidad Montero-Vilchez, María I. Quiñones-Vico, Manuel Sanchez-Diaz, Salvador Arias-Santiago

**Affiliations:** ^1^Cell Production and Tissue Engineering Unit, Andalusian Network of Design and Translation of Advanced Therapies, Virgen de las Nieves University Hospital, Granada, Spain; ^2^Biosanitary Institute of Granada (ibs.GRANADA), Granada, Spain; ^3^Department of Dermatology, Virgen de las Nieves University Hospital, Granada, Spain; ^4^Department of Dermatology, Faculty of Medicine, University of Granada, Granada, Spain

**Keywords:** advanced therapy, cell therapy, dermatology, mesenchymal stem cells, skin diseases, skin injuries, stem cells, tissue engineering

## Abstract

Skin disease may be related with immunological disorders, external aggressions, or genetic conditions. Injuries or cutaneous diseases such as wounds, burns, psoriasis, and scleroderma among others are common pathologies in dermatology, and in some cases, conventional treatments are ineffective. In recent years, advanced therapies using human mesenchymal stem cells (hMSCs) from different sources has emerged as a promising strategy for the treatment of many pathologies. Due to their properties; regenerative, immunomodulatory and differentiation capacities, they could be applied for the treatment of cutaneous diseases. In this review, a total of thirteen types of hMSCs used as advanced therapy have been analyzed, considering the last 5 years (2015–2020). The most investigated types were those isolated from umbilical cord blood (hUCB-MSCs), adipose tissue (hAT-MSCs) and bone marrow (hBM-MSCs). The most studied diseases were wounds and ulcers, burns and psoriasis. At preclinical level, *in vivo* studies with mice and rats were the main animal models used, and a wide range of types of hMSCs were used. Clinical studies analyzed revealed that cell therapy by intravenous administration was the advanced therapy preferred except in the case of wounds and burns where tissue engineering was also reported. Although in most of the clinical trials reviewed results have not been posted yet, safety was high and only local slight adverse events (mild nausea or abdominal pain) were reported. In terms of effectiveness, it was difficult to compare the results due to the different doses administered and variables measured, but in general, percentage of wound’s size reduction was higher than 80% in wounds, Psoriasis Area and Severity Index and Severity Scoring for Atopic Dermatitis were significantly reduced, for scleroderma, parameters such as Modified Rodnan skin score (MRSC) or European Scleroderma Study Group activity index reported an improvement of the disease and for hypertrophic scars, Vancouver Scar Scale (VSS) score was decreased after applying these therapies. On balance, hMSCs used for the treatment of cutaneous diseases is a promising strategy, however, the different experimental designs and endpoints stablished in each study, makes necessary more research to find the best way to treat each patient and disease.

## Introduction

Human mesenchymal stem cells (hMSCs) are non-hematopoietic multipotent adult progenitor cells that are found in multiple tissues. They can be easily harvested and expanded from the different tissues of adult donors, avoiding any potential ethical issues for the development of new therapies (Mushahary et al., 2018; Sierra-Sanchez et al., 2018).

The use of hMSCs for dermatological diseases seems to be interesting due to (1) their hypo-immunogenic properties, which allows its immediate use as prepared allogeneic cells without significant host reaction (Koppula et al., 2009; Liang et al., 2010; Lin and Hogan, 2011; Squillaro et al., 2016); (2) their anti-inflammatory capacity (Di Nicola et al., 2002), that can also be useful in dampening the inflammatory milieu of chronic non-healing wounds and aid in the healing process, as well as for the treatment of inflammatory chronic cutaneous diseases; and (3) their possibility to differentiate into both mesenchymal and non-mesenchymal lineages such as ectodermal keratinocyte-like cells (KLCs) (Seo et al., 2016) and dermal cells (He et al., 2007).

In addition, together with adult skin cells and skin stem cells, the role of hMSCs in normal wound healing is also important. They can contribute to re-epithelization by stimulating collagen production and reducing fibrosis and scar formation by releasing many growth factors such as epidermal growth factor (EGF), transforming growth factor beta (TGF-β), vascular endothelial growth factor (VEGF), and basic fibroblast growth factor (bFGF) (Stone Ii et al., 2018).

**FIGURE 1:**
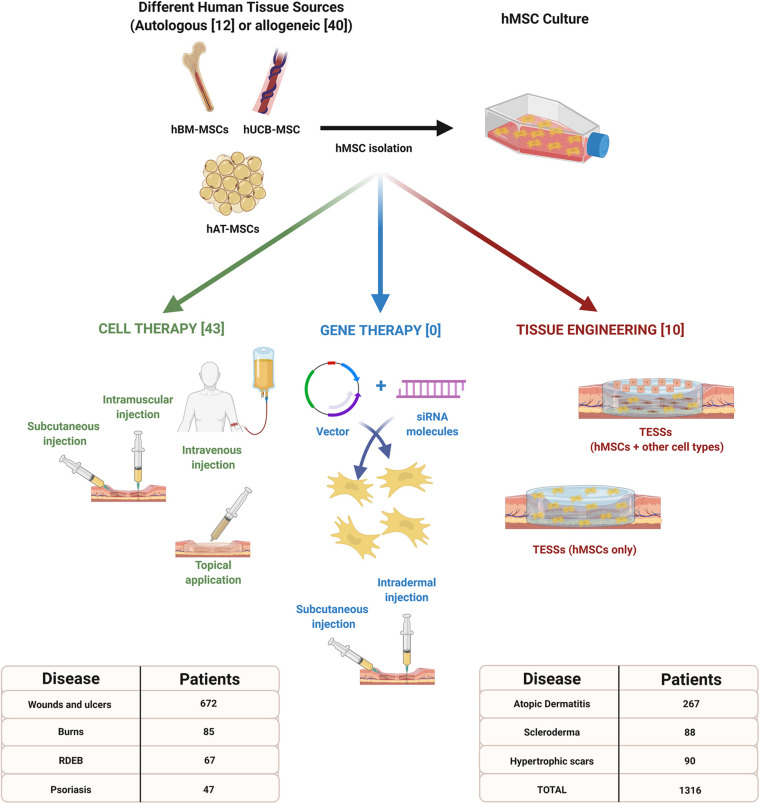
Advanced therapy strategies based on the use of human mesenchymal stem cells (hMSCs) for the treatment of different cutaneous injuries or diseases. Information included in brackets refers to the number of clinical studies reviewed between 2015 and 2020. This infographic also indicates the total number of patients expected to be recruited for each pathology. Created with Biorender.com.

For these reasons, the development and research of hMSC based strategies in dermatology have increased in recent years (Figure 1). The objective of this review is to summarize the recent treatments (from 2015 to 2020) based on the use of hMSCs, under research or applied in patients, to treat the most common cutaneous injuries or diseases.

## Literature Search Methodology

A literature search was performed using PubMed^®^ and ClinicalTrials.gov from 01/01/2015 to 31/12/2020. The following search terms were used: [(Mesenchymal Stem Cell) OR (Mesenchymal Stromal Cell)] AND [(Wound Healing) OR (Skin Wounds) OR (Skin Ulcers) OR (Burns) OR (Recessive Dystrophic Epidermolysis Bullosa) OR (Psoriasis) OR (Atopic Dermatitis) OR (Scleroderma) OR (Hypertrophic Scars) OR (Skin Scars)].

### Inclusion and Exclusion Criteria

The search in PubMed^®^ was limited to: (i) studies using hMSCs, (ii) used as advanced therapy for skin conditions, (iii) in animals or humans, and (iv) written in English or Spanish.

The search in ClinicalTrials.gov was limited to studies where recruitment status was: (Recruiting) OR (Not yet recruiting) OR (Active, not recruiting) OR (Completed) OR (Enrolling by invitation) OR (Suspended) OR (Terminated). Studies where recruitment status was (Withdrawn) OR (Unknown) were excluded.

Reviews, guidelines, protocols, and conference abstracts were excluded.

## Mesenchymal Stem Cells in Cutaneous Injuries or Diseases

### Wounds and Ulcers

Wound healing is a complex but well-orchestrated process divided in four overlapping phases (hemostasis, inflammation, proliferation, and remodeling) which plays a crucial role after a cutaneous injury, restoring function and appearance of damaged skin with minimal scarring (Reinke and Sorg, 2012). However, many factors and diseases can provoke a deregulation of the healing process, manifested as delayed wound healing (diabetes and radiation exposure) or excessive healing (hypertrophic scars) (Gurtner et al., 2008).

#### Preclinical *in vivo* Studies

At preclinical level (Table 1), since 2015, fourteen studies have analyzed the use of hMSCs for wound healing therapies in mouse or rat models. These cells have been isolated from different human tissues such as adipose tissue (hAT-MSCs) (Li et al., 2016; Hersant et al., 2019; Xiao et al., 2019; Zomer et al., 2020), bone marrow (hBM-MSCs) (He et al., 2019), umbilical cord blood (hUCB-MSCs) (Montanucci et al., 2017; Yang et al., 2017; Xu et al., 2019; Myung et al., 2020; Zhang et al., 2020), Wharton’s Jelly (hWJ-MSCs) (Ertl et al., 2018; Millán-Rivero et al., 2019), dermal papilla (hDP-MSCs) (Zomer et al., 2020), placenta (hP-MSCs) (Ertl et al., 2018), menstrual fluid (hMen-MSCs) (Cuenca et al., 2018), amnion (hA-MSCs) (Ertl et al., 2018), and jaw bone marrow (hJM-MSCs) (He et al., 2019). In one study the human source of hMSCs was not indicated (Chen et al., 2017). First conclusion is that all treatments based on hMSCs reported better results in terms of wound healing comparing with non-advanced or control therapies. Quantitave comparison of the effectiveness of the different hMSCs populations was difficult because in some cases the numeric information was not provided and also, the follow-up differed.

**TABLE 1:** Preclinical *in vivo* studies of hMSCs used as advanced therapy for wounds and ulcers in the last 5 years.

Type of advanced therapy	Cells	Type of hMSC treatment evaluated	Type of wound analyzed	Follow-up	References
Cell therapy	hAT-MSCs	1-hAT-MSC + SDF-1: intradermal injection around the wound bed of 10^6^ cells pretreated with SDF-1 2-hAT-MSC: intradermal injection around the wound bed of 10^6^ cells	Diabetic chronic skin wounds	10 days	Li et al., 2016
	hMen-MSCs	1-hMen-MSC group: intradermal injections around each wound of 10^6^ cells	Excisional wounds	14 days	Cuenca et al., 2018
	hAT-MSCs	1-hAT-MSC from non-diabetic patients: subcutaneous injection of 10^6^ cells 2-hAT-MSC from diabetic patients: subcutaneous injection of 10^6^ cells	Pressure ulcer wounds	21 days	Xiao et al., 2019
	hBM-MSCs and hJM-MSCs	1-hBM-MSC: intravenous injection of 2 × 10^6^ cells/mL 2-hJM-MSC: intravenous injection of 2 × 10^6^ cells/mL	Full-thickness skin excision	12 days	He et al., 2019
	hAT-MSCs	1-hAT-MSC: Injection in the wound of 2 × 10^5^ cells 2-hAT-MSC + Plasma: Injection in the wound of 2 × 10^5^ cells with 20% of PRP activated with 10% CaCl_2_	Full-thickness skin excision	10 days	Hersant et al., 2019
	hUCB-MSCs	1-hUCB-MSC: Injection in the wound of 5 × 10^6^ cells 2-hUCB-MSC + Hydrogel: Injection in the wound of 5 × 10^6^ cells with a thermo-sensitive gel	Incision wounds	21 days	Xu et al., 2019
	hUCB-MSCs	1-Irradiated wound treated with hUCB-MSCs: Implantation onto the wound bed of 1 × 10^5^ cells in 30 μL Matrigel and subcutaneous injection around the wound of 4 × 10^5^ cells in 120 μL Matrigel 2-Irradiated wound treated with hUCB-MSCs and PRP: Implantation onto the wound bed of 1 × 10^5^ cells in 30 μL PRP and subcutaneous injection around the wound of 4 × 10^5^ cells in 120 μL PRP	Combined radiation and wound injury	21 days	Myung et al., 2020
	hUCB-MSCs	1-hUCB-MSC: subcutaneous injection around the wounds of 10^6^ cells	Diabetic skin wounds	14 days	Zhang et al., 2020
Cell therapy and tissue engineering	hWJ-MSCs	1-Injected hWJ-MSC: injection of 10^6^ cells in 100 μl buffered saline solution 2-Skin substitute: wounds covered by cellularized silk fibroin scaffold (5 × 10^4^ hWJ-MSC seeded onto the scaffold for 4 days before surgery) 3-Combined therapy: wounds treated with hWJ-MSCs injected at the edge (10^6^) and also cellularized silk fibroin patches (5 × 10^4^ hWJ-MSCs seeded onto the scaffold for 4 days before surgery)	Excisional wounds	28 days	Millán-Rivero et al., 2019
Tissue engineering	hUCB-MSCs	1-Dermal equivalent (DE): Fibrin based scaffold mixed with 4 × 10^5^ cells + 6 × 10^5^ cells 2-Scaffold (S): Fibrin based scaffold mixed with 4 × 10^5^ cells	Full-Thickness Lesions	36 days	Montanucci et al., 2017
	hMSCs (not defined)	1-h-MSC group: h-MSC cell sheets constituted of 10,000 cells/cm^2^ + Autograft 2-Prevascularized h-MSC group: h-MSC cell sheets constituted of 10,000 cells/cm^2^ + 20,000 HUVEC cells/cm^2^ on top + Autograft	Full thickness excision wounds	28 days	Chen et al., 2017
	hUCB-MSCs	1- hUCB-MSCs + Plasma: Platelet poor plasma gel combined with amnion (PPPA) + 10^6^ hUCB-MSCs 2- hUCB-MSCs injected: 10^6^ hUCB-MSCs injected subcutaneously	Full-thickness excisional skin wounds	14 days	Yang et al., 2017
	hA-MSCs, hP-MSCs and hWJ-MSCs	1-hMSC: Matriderm^®^ + 3 × 10^5^ cells (hA-MSCs alone, hP-MSCs alone or hWJ-MSCs alone) 2-Prevascularized + hMSCs: Matriderm^®^ + PLECs mixed with the respective hMSC type in an established 80:20 ratio	Full-thickness wounds	8 days	Ertl et al., 2018
	hDP-MSCs and hAT-MSCs	1-hDP-MSC: engrafment of Integra^®^ associated with 10^6^ cells 2-hAT-MSC: engrafment of Integra^®^ associated with 10^6^ cells	Excisional wounds	60 days	Zomer et al., 2020

##### Cell therapy

Apart from the type of hMSCs, these studies were organized according to the advanced therapy analyzed in each case (Table 1). Most of the research used hMSCs as cell therapy (CT) (Li et al., 2016; Cuenca et al., 2018; He et al., 2019; Hersant et al., 2019; Xiao et al., 2019; Xu et al., 2019; Myung et al., 2020; Zhang et al., 2020). Mean follow-up for this strategy was 15.4 ± 4.9 days but routes of administration varied from direct application onto the wounds (Hersant et al., 2019; Xu et al., 2019; Myung et al., 2020), intradermal injection (Li et al., 2016; Cuenca et al., 2018), and subcutaneous injection (Xiao et al., 2019; Zhang et al., 2020) to intravenous injection (He et al., 2019).

hAT-MSCs were used in three studies (Li et al., 2016; Hersant et al., 2019; Xiao et al., 2019). Li et al. (2016) compared the use of hAT-MSCs (10^6^ cells), hAT-MSCs (10^6^ cells) pretreated with stromal cell-derived factor 1 (SDF-1) and control group, demonstrating that SDF-1 provided a protective effect on hMSCs survival and, moreover, that increased wound closure potential of diabetic chronic wounds. Xiao et al. (2019) analyzed the diabetic or non-diabetic origin of hAT-MSCs, and their potential for pressure ulcer wounds treatment, reporting that both populations (10^6^ cells of each) suppressed inflammatory response and improved wound skin regeneration but wound healing was better in the case of non-diabetic hAT-MSCs. Hersant et al. (2019) studied the use of hAT-MSCs alone (2 × 10^5^ cells) or combined with 20% platelet-rich plasma (PRP) revealing that wound closure were significantly higher in the combined therapy, with more expression of human VEGF and SDF-1.

The another preferred hMSC population for these studies were hUCB-MSCs, which were investigated in three articles also (Xu et al., 2019; Myung et al., 2020; Zhang et al., 2020). Xu et al. (2019) compared the use of hUCB-MSCs alone (5 × 10^6^ cells) or in combination with an hydrogel, however, significant differences in terms of wound healing were only observed against control treatment groups without cells. Myung et al. (2020) design a similar experiment but using PRP instead of and hydrogel. In this case, the number of hUCB-MSCs used was 10^5^ cells and, only combined therapy (hUCB-MSCs + PRP) significantly accelerated wound closure and enhanced neovascularization. Interestingly, Zhang et al. (2020) analyzed the use of hUCB-MSCs (10^6^ cells), medium derived from hMSC cultures and human fibroblasts (10^6^ cells), demonstrating that hUCB-MSCs or culture medium treatments accelerated wound healing by enhancing angiogenesis.

Remaining studies which reported the use of hMSCs as CT only, analyzed lesser-used sources such as hMen-MSCs (Cuenca et al., 2018) or hJM-MSCs (He et al., 2019). In the first case, 10^6^ hMen-MSCs were intradermally injected and compared with a control treatment group demonstrating that wound closure was higher and a well-defined vascular network was promoted by hMen-MSCs (Cuenca et al., 2018). He et al. (2019) analyzed the intravenous injection of hBM-MSCs (2 × 10^6^ cells/mL) or hJM-MSCs (2 × 10^6^ cells/mL) reporting better results in terms of wound closure against control group, but without significant differences between both hMSCs sources.

##### Tissue engineering

Tissue engineering (TE) is another type of advanced therapy which has been investigated for the treatment of wounds and ulcers (Table 1). From 2015, five studies have reported the use of hMSCs to evaluate their potential clinical benefits as tissue-engineered skin substitute (TESS) (Chen et al., 2017; Montanucci et al., 2017; Yang et al., 2017; Ertl et al., 2018; Zomer et al., 2020). Mean time of follow-up for this strategy was 29.2 ± 20.4 days and the hMSCs populations analyzed were hUCB-MSCs (Montanucci et al., 2017; Yang et al., 2017), hA-MSCs, hP-MSCs, hWJ-MSCs (Ertl et al., 2018), hDP-MSCs, hAT-MSCs (Zomer et al., 2020), and one where the source was not indicated (Chen et al., 2017).

In the case of hUCB-MSCs, different strategies were analyzed. Montanucci et al. (2017) reported their use as part of a fibrin-based scaffold only (4 × 10^5^ cells) or culturing hUCB-MSCs over this scaffold (6 × 10^5^ cells). These substitutes were transplanted onto full-thickness lesions, and wounds treated with both cell layers seemed to heal slower than those treated with fibrin-based scaffold only, although the wound outcome at the end of the study looked much better in the first case. Yang et al. (2017) also administered hUCB-MSCs and compared their use as part of a TESS constituted of platelet poor plasma gel, amnion and 10^6^ cells or as CT (10^6^ cells injected subcutaneously). Results revealed that thickness of the newly formed epidermis layer of the TESS group grew faster to cover the wounded skin tissue than the CT and control groups.

Ertl et al. (2018) compared different hMSCs sources (hA-MSCs, hP-MSCs, hWJ-MSCs) cultured over Matriderm^®^ alone (3 × 10^5^ cells) or in combination with placental endothelial cells (PLECs). Interestingly, single application of each hMSC type induced a better wound reduction than the co-applications with PLECs. The best results in terms of wound healing were reported by the use of hA-MSCs. Zomer et al. (2020) also compared two types of hMSCs (hDP-MSCs, hAT-MSCs – 10^6^ cells) associated to Integra^®^, demonstrating that these groups presented significantly higher closure than control group (Integra^®^).

Finally, Chen et al. (2017) evaluated the use of hMSCs sheets (10^4^ cells/cm^2^) pre-vascularized or not, as a support treatment for gold standard therapy (autografts). Grafts from pre-vascularized group preserved most skin appendages and supporting loose connective tissues and on balance, transplantation of autografts with hMSCs significantly accelerated wound healing.

##### Combined advanced therapy: cell therapy and tissue engineering

Interestingly, one study evaluated a combined therapy of cell therapy and tissue engineering (Table 1). In this research, hWJ-MSCs were injected (10^6^ cells) around the wounds, cultured over a silk fibroin scaffold and transplanted (5 × 10^4^ cells) or both treatments were evaluated together. Results revealed that combined therapy group displayed a collagen dermis organization that was more similar to that typically observed in the normal skin of mice and exhibited better wound healing capabilities as compared with both single treatments (Millán-Rivero et al., 2019).

##### Brief conclusion

On balance, potential benefits of these wound healing treatments have been evaluated for four types of skin injuries: diabetic chronic wounds (Li et al., 2016; Zhang et al., 2020), excisional wounds (Cuenca et al., 2018; Ertl et al., 2018; He et al., 2019; Hersant et al., 2019; Millán-Rivero et al., 2019; Xu et al., 2019; Zomer et al., 2020), pressure ulcers wounds (Xiao et al., 2019), and combined radiation and wound injuries (Myung et al., 2020). Results revealed that both hMSCs’ strategies, CT or TE, reported better results in terms of re-epithelialization, wound closure and vascularization than control and other treatment groups (autograft, injection of buffered saline solution, or hydrogel without cells) (Table 1).

#### Clinical Studies

Considering the use of hMSCs for clinical purposes, sixteen studies or clinical trials have been reviewed (Table 2). According to the preclinical *in vivo* studies, advanced therapies preferred have been cell therapy (8 studies) and tissue engineering (8 studies). The main source of hMSCs analyzed was hAT-MSCs (7) although hWJ-MSCs (2), hUCB-MSCs (2), hBM-MSCs (1), hP-MSCs (1), and non-indicated source of hMSCs (3) were also analyzed. Considering the use of autologous or allogeneic cells, allogeneic source was applied in most of the studies (14).

**TABLE 2:** Clinical studies of hMSCs used as advanced therapy for wounds and ulcers in the last 5 years.

Type of advanced therapy	Cells	Type of clinical study	N (male/female)	Age (years)^a^	hMSC treatment	Safety (Treatment-related adverse events)	Indication	Affected area^a^	Effectiveness^a^ (wound’s size reduction)	Follow-up^a^	References
Cell Therapy	Allogeneic hBM-MSCs	Case Report	1 (1/0)	66	hBM-MSCs at a concentration of 10^6^ cells/ml was injected around and within the lesion	None	Chronic radiation-induced skin lesion	48 cm^2^	100%	2 years	Portas et al., 2016
	Allogeneic hWJ-MSCs	Case Report	1 (0/1)	41	Intradermal injection around and within the lesion of 10^5^ cells/cm^2^	None	Chronic ulcers	42 cm^2^	75%	14 days	Mejía-Barradas et al., 2019
	Allogeneic hUCB-MSCs	Phase I Randomized Clinical Trial (Parallel Assignment-Single Blind)	110	(8–12)	Skin grafting and application of hUCB-MSCs (injection)	–	Traumatic heel pad injuries	–	No results posted	90 days	NCT04219657 (Comparison Between Skin Graft Versus Skin Graft and Stem Cell Application – Full Text View – ClinicalTrials.gov)^10^
	Allogeneic hMSCs (not defined)	Phase I/IIa Multicenter Clinical Trial (Single Group Assignment-Open Label)	31	(35–85)	hMSCs applied on the wound surface on Days 0 and Week 6	–	Chronic venous leg ulcers	–	No results posted	12 months	NCT03257098 (Allogeneic ABCB5-positive Stem Cells for Treatment of CVU – Full Text View – ClinicalTrials.gov)^2^
	Autologous hMSCs (not defined)	Phase I/IIa Clinical Trial (Single Group Assignment-Open Label)	13	(18–85)	Topical application of hMSCs on the wound surface	–	Chronic venous leg ulcers	–	No results posted	12 months	NCT02742844 (Clinical Trial to Investigate Efficacy and Safety of the IMP in Patients With Non-Healing Wounds Originating From Ulcers – Full Text View – ClinicalTrials.gov)^9^
	Allogeneic hUCB-MSCs	Phase I Non- Randomized Clinical Trial (Sequential Assignment-Open Label)	20	Older than 18	3 doses of expanded allogeneic hUCB-MSCs	–	Diabetic foot ulcers	–	No results posted	4 months	NCT04104451 (PHASE 1, OPEN-LABEL SAFETY STUDY OF UMBILICAL CORD LINING MESENCHYMAL STEM CELLS (CORLICYTE) TO HEAL CHRONIC DIABETIC FOOT ULCERS – Full Text View – ClinicalTrials.gov)^12^
	Allogeneic hMSCs (not defined)	Phase I/IIa Multicenter Clinical Trial (Single Group Assignment-Open Label)	23	(18–85)	Two doses of allogeneic hMSCs on patients wound	–	Diabetic foot ulcers	–	No results posted	12 months	NCT03267784 (Allogeneic ABCB5-positive Stem Cells for Treatment of DFU “Malum Perforans” – Full Text View – ClinicalTrials.gov)^3^
	Allogeneic hP-MSCs	Phase I Non- Randomized Clinical Trial (Single Group Assignment-Open Label)	43	(18–75)	Single dose of hP-MSCs gel on the wound or multidose on six consecutive days	–	Diabetic foot ulcers	–	No results posted	34 days	NCT04464213 (Human Placental Mesenchymal Stem Cells Treatment on Diabetic Foot Ulcer – Full Text View – ClinicalTrials.gov)^11^
Tissue Engineering	Allogeneic hWJ-MSCs	Randomized Clinical Trial	5	(30–60)	Acellular amniotic membrane seeded with hWJ-MSCs	None	Chronic diabetic wounds	0.71 cm^2^	96.7%	30 days	Hashemi et al., 2019
	Allogeneic hAT-MSCs	Phase II Multicenter Randomized Clinical Trial (Parallel Assignment-Single Blind)	22 (14/8) 17 (13/4) – Control Group	59.9 ± 13.3 (26–80) 68.4 ± 9.9 (43–79)	Hydrogel sheet containing 10^6^ hAT-MSCs	No serious adverse events were observed	Diabetic foot ulcers	1–25 cm^2^	Complete wound closure was achieved for 82% of patients in the treatment group and 53% in the control group at week 12	12 weeks	NCT02619877 (Clinical Study to Evaluate Efficacy and Safety of ALLO-ASC-DFU in Patients With Diabetic Foot Ulcers – Full Text View – ClinicalTrials.gov)^7^ (Moon et al., 2019)
	Autologous hAT-MSCs	Prospective clinical analysis	6 (3/3)	66.3 ± 9.0	Bio-membranes constituted of 10^7^ hAT-MSCs + platelet-rich plasma applied topically on each ulcer	None	Chronic diabetic ulcers	6.7 cm^2^	74.5 ± 32.5%	90 days	Stessuk et al., 2020
	Allogeneic hAT-MSCs	Phase II Randomized Clinical Trial (Parallel Assignment-Quadruple Blind)	64	(18–80)	Hydrogel sheet containing hAT-MSCs	–	Diabetic foot ulcers	–	No results posted	36 weeks	NCT04497805 (Clinical Study of ALLO-ASC-SHEET in Subjects With Diabetic Wagner Grade II Foot Ulcers – Full Text View – ClinicalTrials.gov)^5^
	Allogeneic hAT-MSCs	Phase III Multicenter Randomized Clinical Trial (Parallel Assignment-Double Blind)	164	(18–75)	Hydrogel sheet containing hAT-MSCs	–	Diabetic Foot ulcers	–	No results posted	12 weeks	NCT03370874 (Clinical Study to Evaluate Efficacy and Safety of ALLO-ASC-DFU in Patients With Diabetic Foot Ulcers. – Full Text View – ClinicalTrials.gov)^6^
	Allogeneic hAT-MSCs	Phase II Randomized Clinical Trial (Parallel Assignment-Double Blind)	44	(18–80)	Hydrogel sheet containing hAT-MSCs	–	Diabetic foot ulcers	–	No results posted	36 weeks	NCT03754465 (Clinical Study of ALLO-ASC-SHEET in Subjects With Diabetic Foot Ulcers – Full Text View – ClinicalTrials.gov)^4^
	Allogeneic hAT-MSCs	Observational Sutdy of Phase I Clinical Trial	4	(18–80)	Hydrogel sheet containing hAT-MSCs	–	Diabetic foot ulcers	–	No results posted	24 months	NCT03183726 (A Follow-up Study to Evaluate the Safety of ALLO-ASC-DFU in ALLO-ASC-DFU-101 Clinical Trial – Full Text View – ClinicalTrials.gov)^1^
	Allogeneic hAT-MSCs	Phase III Multicenter Randomized Clinical Trial (Parallel Assignment-Double Blind)	104	(19–75)	Hydrogel sheet containing allogeneic hAT-MSCs	–	Diabetic foot ulcers	–	No results posted	12 weeks	NCT04569409 (Clinical Study to Evaluate Efficacy and Safety of ALLO-ASC-DFU in Patients With Diabetic Wagner Grade 2 Foot Ulcers. – Full Text View – ClinicalTrials.gov)^8^

*^*a*^Expression of measures: mean ± standard deviation (range).^1^A Follow-up Study to Evaluate the Safety of ALLO-ASC-DFU in ALLO-ASC-DFU-101 Clinical Trial – Full Text View – ClinicalTrials.gov Available at: https://clinicaltrials.gov/ct2/show/NCT03183726 [Accessed January 13, 2021].^2^Allogeneic ABCB5-positive Stem Cells for Treatment of CVU – Full Text View – ClinicalTrials.gov Available at: https://www.clinicaltrials.gov/ct2/show/NCT03257098?term=mesenchymal+stem+cells&cond=Wound+of+Skin&strd_s=01%2F01%2F2015&strd_e=12%2F31%2F2020&draw=2&rank=4 [Accessed January 11, 2021].^3^Allogeneic ABCB5-positive Stem Cells for Treatment of DFU “Malum Perforans” – Full Text View – ClinicalTrials.gov Available at: https://www.clinicaltrials.gov/ct2/show/study/NCT03267784?term=mesenchymal+stem+cells&cond=Skin+ulcer&strd_s=01%2F01%2F2015&strd_e=12%2F31%2F2020&draw=2&rank=19 [Accessed January 13, 2021].^4^Clinical Study of ALLO-ASC-SHEET in Subjects With Diabetic Foot Ulcers – Full Text View – ClinicalTrials.gov Available at: https://www.clinicaltrials.gov/ct2/show/study/NCT03754465?term=mesenchymal+stem+cells&cond=Skin+ulcer&strd_s=01%2F01%2F2015&strd_e=12%2F31%2F2020&draw=2&rank=13 [Accessed January 13, 2021].^5^Clinical Study of ALLO-ASC-SHEET in Subjects With Diabetic Wagner Grade II Foot Ulcers – Full Text View – ClinicalTrials.gov Available at: https://www.clinicaltrials.gov/ct2/show/study/NCT04497805?term=mesenchymal+stem+cells&cond=Skin+ulcer&strd_s=01%2F01%2F2015&strd_e=12%2F31%2F2020&draw=2&rank=10 [Accessed January 13, 2021].^6^Clinical Study to Evaluate Efficacy and Safety of ALLO-ASC-DFU in Patients With Diabetic Foot Ulcers. – Full Text View – ClinicalTrials.gov Available at: https://www.clinicaltrials.gov/ct2/show/study/NCT03370874?term=mesenchymal+stem+cells&cond=Skin+ulcer&strd_s=01%2F01%2F2015&strd_e=12%2F31%2F2020&draw=2&rank=12 [Accessed January 13, 2021].^7^Clinical Study to Evaluate Efficacy and Safety of ALLO-ASC-DFU in Patients With Diabetic Foot Ulcers – Full Text View – ClinicalTrials.gov Available at: https://www.clinicaltrials.gov/ct2/show/study/NCT02619877?term=mesenchymal+stem+cells&cond=Skin+ulcer&strd_s=01%2F01%2F2015&strd_e=12%2F31%2F2020&draw=2&rank=11 [Accessed January 13, 2021].^8^Clinical Study to Evaluate Efficacy and Safety of ALLO-ASC-DFU in Patients With Diabetic Wagner Grade 2 Foot Ulcers. – Full Text View – ClinicalTrials.gov Available at: https://www.clinicaltrials.gov/ct2/show/NCT04569409?term=mesenchymal+stem+cells&cond=Skin+ulcer&strd_s=01%2F01%2F2015&strd_e=12%2F31%2F2020&draw=2&rank=21 [Accessed January 13, 2021].^9^Clinical Trial to Investigate Efficacy and Safety of the IMP in Patients With Non-Healing Wounds Originating From Ulcers – Full Text View – ClinicalTrials.gov Available at: https://www.clinicaltrials.gov/ct2/show/NCT02742844?term=mesenchymal+stem+cells&cond=Wound+of+Skin&strd_s=01%2F01%2F2015&strd_e=12%2F31%2F2020&draw=1&rank=6 [Accessed January 11, 2021].^10^Comparison Between Skin Graft Versus Skin Graft and Stem Cell Application – Full Text View – ClinicalTrials.gov Available at: https://www.clinicaltrials.gov/ct2/show/study/NCT04219657?term=mesenchymal+stem+cells&cond=Wound+of+Skin&strd_s=01%2F01%2F2015&strd_e=12%2F31%2F2020&draw=2&rank=1 [Accessed January 11, 2021].^11^Human Placental Mesenchymal Stem Cells Treatment on Diabetic Foot Ulcer – Full Text View – ClinicalTrials.gov Available at: https://www.clinicaltrials.gov/ct2/show/NCT04464213?term=mesenchymal+stem+cells&cond=Skin+ulcer&strd_s=01%2F01%2F2015&strd_e=12%2F31%2F2020&draw=2&rank=3 [Accessed January 11, 2021].^12^PHASE 1, OPEN-LABEL SAFETY STUDY OF UMBILICAL CORD LINING MESENCHYMAL STEM CELLS (CORLICYTE^®^) TO HEAL CHRONIC DIABETIC FOOT ULCERS – Full Text View – ClinicalTrials.gov Available at: https://www.clinicaltrials.gov/ct2/show/study/NCT04104451?term=mesenchymal+stem+cells&cond=Skin+ulcer&strd_s=01%2F01%2F2015&strd_e=12%2F31%2F2020&draw=2&rank=6 [Accessed January 13, 2021].*

##### Cell therapy

The use of hMSCs as CT for the treatment of wounds and ulcers is an interesting alternative against conventional therapies. In this sense, from 2015 to 2020, eight studies have reported their use (Table 2): two of them were case reports and results of efficiency, in terms of wound’s size reduction, were published (Portas et al., 2016; Mejía-Barradas et al., 2019).

Portas et al. (2016) evaluated the injection of 10^6^ allogeneic hBM-MSCs/ml around and within chronic radiation-induced skin lesion of one patient (66 years old). After 3 months, wound size was completely reduced without non-adverse events. Moreover, the use of hBM-MSCs reduced inflammation process (marked decrease of β1 integrin expression on lymphocytes) and improved vasculature and quality of the skin.

Mejía-Barradas et al. (2019) studied the intradermal injection around and within the lesion of 10^5^ allogeneic hWJ-MSCs/cm^2^, in one patient (41 years old) with chronic ulcers (42 cm^2^) reporting an effectiveness of 75%, considering wound size reduction after 14 days. Neo-vascularization and formation of collagen fibers were also observed in regenerated skin and the number of proinflammatory cytokines decreased.

Rest of studies reviewed were clinical trials (Phase I or Phase I/IIa), however, no published results are posted at this time. One of them analyzed the topical application of autologous hMSCs on the wound surface for the treatment of chronic venous leg ulcers (NCT02742844), meanwhile, the rest evaluated allogeneic cells such as hUCB-MSCs (NCT04219657 and NCT04104451), hP-MSCs (NCT04464213), or hMSCs (NCT03257098 and NCT03267784) for the treatment of different types of wounds (diabetic foot ulcers, traumatic heel pad injuries and chronic venous leg ulcers).

Considering protocols approved for all these studies, there is not a preferred source of hMSCs for the development of cell therapy strategies for wounds and ulcers, although in all cases, cells will be applied or injected around and into the wounds to evaluate their potential clinical safety and effect.

##### Tissue engineering

From 2015, eight studies have evaluated the use of hMSCs as a component of TESSs for the treatment of diabetic foot ulcers (Table 2). Until now, only three of them have published results of safety and effectiveness (Hashemi et al., 2019; Moon et al., 2019; Stessuk et al., 2020).

Seven studies evaluated the use of allogeneic hMSCs isolated from adipose tissue (6) or Wharton’s Jelly (1) and one study reported the use of autologous hAT-MSCs. Most of them were Phase I, II, or III clinical trials although one of them was an observational study (NCT03183726).

In all cases, strategy was based on manufacturing sheets of different biomaterials combined with the different hMSCs sources to evaluate their engraftment into diabetic foot ulcers. Those studies with published results applied different hMSCs: allogeneic hWJ-MSCs (Hashemi et al., 2019), allogeneic hAT-MSCs (Moon et al., 2019) and autologous hAT-MSCs (Stessuk et al., 2020).

Hashemi et al. (2019) evaluated the engraftment of an acellular amniotic membrane seeded with hWJ-MSCs in 5 patients. No adverse events were reported, and wound size after 9 days significantly declined (96.7% from original size).

Moon et al. (2019) (NCT02619877) manufactured hydrogel sheets containing 10^6^ allogeneic hAT-MSCs/sheet for the treatment of 22 patients with diabetic foot ulcers and compared the results with a standard treatment (17 patients). Outcomes revealed that no serious adverse events were observed, complete wound closure was achieved for 82% in the treatment group and 53% in the control group at week 12 and Kaplan-Meier median times to complete closure were 28.5 and 63.0 days for the treatment group and the control group, respectively.

In a case where autologous hAT-MSCs were used to fabricate bio-membranes constituted of 10^7^ hAT-MSCs and platelet-rich plasma, 6 patients were treated. Conclusions revealed that there was granulation tissue formation starting from 7 days after topical application and after 90 days, a healed and re-epithelialized tissue was observed. No adverse events were reported (Stessuk et al., 2020).

Remaining studies reviewed have not published results yet (NCT04497805, NCT03370874, NCT03754465, NCT03183726, and NCT04569409), but incorporation of hAT-MSCs into a hydrogel was the methodology selected in all cases.

##### Brief conclusion

On balance, preferred hMSC population for the treatment of wounds and ulcers are the allogeneic hAT-MSCs, mainly as tissue engineering strategy. Although information about the results is limited and the standardization of a cell dose is difficult, the use of hMSCs could improve the treatment of patients with these skin conditions, because published results are hopeful.

Considering only those studies were results were posted from 2015 to 2020, 35 patients have been treated (2 with CT strategy and 33 with TE strategy), all of them with chronic skin ulcers (associated to diabetes or not) and a range of wound size between 0.71 and 48 cm^2^. The weighted mean effectiveness, based on the wound’s size reduction, was of 87.5 ± 12.5% for the CT treatments (closure time: from 7 to 90 days) and 82.7 ± 5.8% for TE therapies (closure time: from 9 to 90 days).

### Burn Injuries

The majority of burn injuries are minor and either do not require treatment or can be treated by any caregiver, however, in the case of severe burns they can lead to a profound systemic response and have serious long-term effects on patients (Porter et al., 2016). Moreover, failure to properly treat these injuries will lead to rapid development of organ failure and death (Greenhalgh, 2019).

#### Preclinical *in vivo* Studies

At preclinical level, five studies [one CT (Pourfath et al., 2018) and four TE strategies (Steffens et al., 2017; Kaita et al., 2019; Mahmood et al., 2019; Nazempour et al., 2020)] have evaluated the use of hMSCs for burn injuries in the last 5 years (Table 3).

**TABLE 3:** Preclinical *in vivo* studies of hMSCs used as advanced therapy for burns in the last 5 years.

Type of Advanced Therapy	Cells	Type of hMSC treatment evaluated	Type of wound analyzed	Follow-up	References
Cell therapy	hWJ-MSCs	1-hWJ-MSC: 5 × 10^5^ cells using cell spray method	Third degree burns	21 days	Pourfath et al., 2018
Tissue engineering	hDT-MSCs	1-hDT-MSCs + poly-D,L-lactic acid (PDLLA) + laminin-332 protein (lam): 10^5^ cells + 10^5^ keratinocytes + scaffold 2- hDT-MSCs + PDLLA: 10^5^ cells + 10^5^ keratinocytes + scaffold	Third degree burns	9 days	Steffens et al., 2017
	hUCB-MSCs	1-hUCB-MSC + plasma: 2.5 × 10^4^ derived fibroblast cells + 3 × 10^4^ derived keratinocyte cells + scaffold	Burn injury	20 days	Mahmood et al., 2019
	hAT-MSCs	1-Fresh hAT-MSCs: 5 × 10^4^ cells on artificial dermis 2-Frozen hAT-MSCs: 5 × 10^4^ cells on artificial dermis	Third degree burn wound	12 days	Kaita et al., 2019
	hWJ-MSCs	1-hWJ-MSC + acellular matrix: 2 × 10^6^ hWJSCs seeded onto acellular dermal matrix scaffold	Third-degree burn	21 days	Nazempour et al., 2020

##### Cell therapy

Pourfath et al. (2018) evaluated a cell spray application of 5 × 10^5^ hWJ-MSCs over third degree burns. After 21 days of follow-up, wounds showed a higher degree of re-epithelialization compared to the control group, and hemorrhage was also completely ceased by the end of the second week post application (Table 3).

##### Tissue engineering

In this case, each of the four studies reported, used a different hMSCs population (Table 3): deciduous teeth (hDT-MSCs) (Steffens et al., 2017), hUCB-MSCs (Mahmood et al., 2019), hAT-MSCs (Kaita et al., 2019) and hWJ-MSCs (Nazempour et al., 2020). The average follow-up of these studies was 15.5 ± 5.9 days and third-degree burns in mice and rats was the preferred model, although the type of TESS evaluated varied.

Steffens et al. (2017) embedded hDT-MSCs into a poly-D, L-lactic acid (PDLLA) scaffold and included 10^5^ keratinocytes. Apart from that, they also evaluated the incorporation, or not, of laminin-332 to the design. Considering average size of the lesions, no statistical difference between the different groups were observed, although those groups which incorporated laminin-332 reported a greater reduction.

Mahmood et al. (2019) analyzed an interesting approach where hUCB-MSCs were *in vitro* differentiated into fibroblasts and keratinocytes. Derived fibroblasts (2.5 × 10^4^ cells) were embedded into a plasma scaffold and then, derived keratinocytes (3 × 10^4^ cells) were overlaid. This was compared with a control group without any treatment, revealing that contraction was 97.6 ± 0.61% for hUCB-MSCs group against 87.57 ± 1.30% in control group and complete healing was faster for hUCB-MSCs group (20.00 ± 2.00 vs. 27.67 ± 2.51 days).

Kaita et al. (2019) studied if differences between fresh hAT-MSCs (5 × 10^4^) or frozen hAT-MSCs (5 × 10^4^) cultured over an artificial dermis exists. Results indicated that after day 12 post treatment, a significant difference in the percentage of wound closure was observed between the hAT-MSCs groups and control group but expression of Type I and III collagen was higher when frozen hAT-MSCs were used.

Finally, Nazempour et al. (2020) evaluated the use of 2 × 10^6^ hWJ-MSCs seeded onto acellular dermal matrix scaffold and compared the results with a conventional treatment (silver sulfadiazine ointment). Wound size decreased in a better way in group where hWJ-MSCs were used (87.6%).

##### Brief conclusion

In conclusion, for burn injuries the purpose is to develop a TESS which could supply the lack of health tissue around the wounds. Although, there is not a preferred hMSCs source and more investigation to find the best scaffold is required, it is clear that the use of advanced therapy could improve the treatment of these injuries, even when frozen cells are used.

#### Clinical Studies

For clinical purposes, six studies or clinical trials have investigated the use of hMSCs for the treatment of burn injuries (Table 4). In contrast to the tendency observed in the case of preclinical *in vivo* studies analyzed, the advanced therapy most studied was CT (4 studies vs. 2 studies in the case of TE). Three sources of hMSCs were applied: hAT-MSCs (3), hBM-MSCs (2), and hUCB-MSCs (2). Allogeneic cells were used in four studies and autologous cells only in one. One clinical trial did not define the use of allogeneic or autologous hAT-MSCs (NCT03686449).

**TABLE 4:** Clinical studies of hMSCs used as advanced therapy for burns in the last five years.

Type of advanced therapy	Cells	Type of clinical study	N (male/female)	Age (years)^a^	hMSC treatment	Safety (treatment-related adverse events)	Indication	Total body surface area (TBSA) affected^a^	Effectiveness^a^ (Wound’s size reduction)	Follow-up^a^	References
Cell therapy	Allogeneic hBM-MSCs	Case Report	1 (1/0)	26	Topical application of 10^6^ cells per 100 cm^2^	None	Severe thermal (flame) burns	60%	—	3 years	Mansilla et al., 2015
	Autologous hBM-MSCs Allogeneic hUCB-MSCs	Case-control Randomized prospective study	Autologous hBM-MSCs 20	23 ± 1.9 (20–27)	2 injections in the burned area (10^5^ hBM-MSC/ml)	25% early complications 45% late complications	Thermal full thickness burns	17 ± 2.94% (12–22)	—	6 months	Abo-Elkheir et al., 2017
			Allogeneic hUCB-MSCs 20	22.9 ± 2.73 (18–29)	Topical application and injection	70% early complications 70% late complications		15.95 ± 2.89% (10–20)			
			Control Group 20	25.3 ± 4.38 (18–35)	Early excision and graft-treated group	50% early complications 95% late complications		18.15 ± 2.87% (15–25)			
	Allogeneic hUCB-MSCs	Case Report	1	—	3 × 10^6^ cells/mL applied topically (4 mL)	Minimal hyperpigmentation and hypertrophic scarring	Severe burn injury	70%	97%	6 years	Jeschke et al., 2019
	hAT-MSCs	Randomized Clinical Trial (Parallel Assignment-Open Label)	33	Older than 18	hAT-MSCs-autologous keratinocyte suspension	–	Burn with full-thickness skin loss	–	No results posted	1 month	NCT03686449 (Autologous Keratinocyte Suspension Versus Adipose-Derived Stem Cell-Keratinocyte Suspension for Post-Burn Raw Area – Full Text View – ClinicalTrials.gov)^3^
Tissue engineering	Allogeneic hAT-MSCs	Phase I Clinical Trial (Single Group Assignment- Open Label)	5	Older than 18	Hydrogel sheet containing allogeneic hAT-MSCs	–	Second-degree burn wounds	–	No results posted	4 weeks	NCT02394873 (A Study to Evaluate the Safety of ALLO-ASC-DFU in the Subjects With Deep Second-degree Burn Wound – Full Text View – ClinicalTrials.gov)^1^
	Allogeneic hAT-MSCs	Observational Study of Phase I Clinical Trial	5	Older than 18	Hydrogel sheet containing allogeneic hAT-MSCs	–	Second-degree burn wounds	–	No results posted	24 months	NCT03183622 (A Follow-up Study to Evaluate the Safety of ALLO-ASC-DFU in ALLO-ASC-BI-101 Clinical Trial – Full Text View – ClinicalTrials.gov)^2^

*^*a*^Expression of measures: mean ± standard deviation (range).*

*^1^A Study to Evaluate the Safety of ALLO-ASC-DFU in the Subjects With Deep Second-degree Burn Wound – Full Text View – ClinicalTrials.gov Available at: https://www.clinicaltrials.gov/ct2/show/NCT02394873?term=mesenchymal+stem+cells&recrs=abdefgh&cond=burns&strd_s=01%2F01%2F2015&strd_e=12%2F31%2F2020&draw=2&rank=5 [Accessed January 13, 2021].*

*^2^A Follow-up Study to Evaluate the Safety of ALLO-ASC-DFU in ALLO-ASC-BI-101 Clinical Trial – Full Text View – ClinicalTrials.gov Available at: https://www.clinicaltrials.gov/ct2/show/study/NCT03183622?term=mesenchymal+stem+cells&recrs=abdefgh&cond=burns&strd_s=01%2F01%2F2015&strd_e=12%2F31%2F2020&draw=2&rank=6 [Accessed January 13, 2021].*

*^3^Autologous Keratinocyte Suspension Versus Adipose-Derived Stem Cell-Keratinocyte Suspension for Post-Burn Raw Area – Full Text View – ClinicalTrials.gov Available at: https://www.clinicaltrials.gov/ct2/show/NCT03686449?term=mesenchymal+stem+cells&recrs=abdefgh&cond=burns&strd_s=01%2F01%2F2015&strd_e=12%2F31%2F2020&draw=2&rank=4 [Accessed January 13, 2021].*

##### Cell therapy

Since 2015, four studies have evaluated the use of CT for the treatment of severe burn thermal (or not) injuries where a full-thickness skin were lost (Table 4). Two research rereviewed were case report (Mansilla et al., 2015; Jeschke et al., 2019) and the other two were clinical trials (Abo-Elkheir et al., 2017) and (NCT03686449) although in the second case, no results were posted.

Mansilla et al. (2015) reported the use of allogeneic hBM-MSCs in one patient (26 years old) by topical application of 10^6^ hBM-MSCs per 100 cm^2^, combined with autologous meshed skin grafting for severe thermal burns. No adverse events were observed and after 35 days of treatment, with two courses of hBM-MSCs, the complete epithelialization of the wounds were too slow. The other case report used allogeneic hUCB-MSCs for the treatment of severe burns injuries of one patient (Total Body Surface Area-TBSA-affected of 70%). 3 × 10^6^ cells/mL were applied topically and results revealed that 97% of wounds were closed with minimal hyperpigmentation and hypertrophic scarring (Jeschke et al., 2019).

One of the clinical trials included, compared the use of a suspension constituted of hAT-MSCs and autologous keratinocytes with other alternative treatments, however, no results were posted (NCT03686449).

Abo-Elkheir et al. (2017) published the results of an interesting clinical trial where compared three types of treatments for thermal full thickness burns: autologous hBM-MSCs (2 injections in the burned area – 1 ml/cm^2^ of 10^5^ hMSC/ml suspension), allogeneic hUCB-MSCs (topical application and injection) and early excision and graft-treated group (*N* = 20 for each group). Safety results revealed that in the case of hBM-MSCs; 25% of cases presented early complications (infection or partial loss of graft) and 45% late complications (hypo- or hyperpigmentation, contracture scar or hypertrophic scar), in the case of hUCB-MSCs; 70% of cases presented early complications and late complications and in the control group; 50% of cases presented early complications and 95% late complications. Percentage of burn extent was significantly reduced in both hMSC groups as compared to early control group.

##### Tissue engineering

Two clinical trials (interventional and observational) evaluated the use allogeneic hAT-MSCs as part of a hydrogel sheet for the treatment of second-degree burn wounds (NCT02394873 and NCT03183622), however, no results have been posted yet (Table 4).

##### Brief conclusion

On balance, at clinical level the preferred hMSC-based advanced therapy is CT in contrast with preclinical studies where the TE is the most investigated. This means that researches are focusing on develop TESS that mimics native skin in a better way, however, more investigation is required and for these reason the translation of CT therapies (which were more studied in the previous years) is more widespread.

Considering all clinical studies reviewed, 42 patients have been recruited to analyze the potential benefits of hMSCs for the treatment of burn injuries. Most of the studies used allogeneic hMSCs but, in some cases results, were not successful (Mansilla et al., 2015), in contrast to one case where the comparison between autologous or allogeneic CT strategy reported that the best wound closure was achieved when autologous hBM-MSCs were used. In the same study, safety parameters revealed that the use of autologous cells was less dangerous for the patient’s health (Abo-Elkheir et al., 2017). However, rest of studies which evaluated the use of allogeneic cells did not report the presence of adverse events. The weighted mean TBSA treated with hMSCs-based CT strategies has been 18.8 ± 10.4%, until now.

### Recessive Dystrophic Epidermolysis Bullosa

Dystrophic epidermolysis bullosa (DEB) is a genetic skin disorder that usually presents at birth. It is due to the presence of pathogenic variants of the gene *COL7A1* and depending on inheritance pattern is divided into two types: dominant dystrophic epidermolysis bullosa (DDEB) and recessive dystrophic epidermolysis bullosa (RDEB). In DDEB, blistering is often mild but nonetheless heals with scarring. In the case of RDEB, clinical disorders are severe including skin fragility manifest by blistering with minimal trauma that heals with milia and scarring, even in in the neonatal period and therefore, the lifetime risk of aggressive squamous cell carcinoma is higher than 90% (Pfendner and Lucky, 2018).

#### Preclinical *in vivo* Studies

Recessive dystrophic epidermolysis bullosa is a rare disease and for this reason the number of studies in the last 5 years is limited (Table 5). In addition, due to their genetic etiology its study requires complex techniques of genetic engineering which long-term effect in humans is yet unknown.

**TABLE 5:** Preclinical *in vivo* and clinical studies of hMSCs used as advanced therapy for recessive dystrophic epidermolysis bullosa in the last 5 years.

PRECLINICAL IN VIVO STUDIES											

Type of Advanced Therapy	Cells		Disease model and Treatments				Animals	Type of wound analyzed		Follow-up	References
Cell therapy	hBM-MSCs		1–8 h after wounding, 2 × 10^6^ hBM-MSCs were injected at the wound edges and this procedure was repeated 48 h after the first injection 2–8 h after wounding, PBS alone were injected at the wound edges and this procedure was repeated 48 h after the first injection				C7-hypomorphic mice and wild-type littermates	Two full thickness skin wounds were incised at the mid-back		12 weeks	Kühl et al., 2015
Tissue engineering, cell therapy and gene therapy	hUCB-MSCs		1-Skin substitute constituted of RDEB fibroblasts mixed with *COL7A1*-transduced MSCs at a 1:1 ratio. Primary RDEB keratinocytes were then seeded on top 2-Skin substitute constituted of RDEB fibroblasts alone. Primary RDEB keratinocytes were then seeded on top 3-Skin substitute constituted of wild type fibroblasts alone. Wild type keratinocytes were then seeded on top 4-After 6–8 weeks mice treated with human skin substitutes received two intradermal injections of 0.5 × 10^6^ engineered MSCs in 50 ul buffered saline solution each or injected with 2 × 10^6^ engineered MSCs in 150 ul buffered saline solution via tail vein. Controls without cells were also injected				7 Immunod- eficient NOD-*scid* IL2Rgamma^*null*^ mice	Full-thickness wound followed by devitalization of mouse skin		8–10 weeks	Petrova et al., 2020

**CLINICAL STUDIES**											

**Type of advanced therapy**	**Cells**	**Type of clinical study**	**N (male/female)**	**Age (years)^a^**	**hMSC treatment**	**Safety (Treatment-related adverse events)**	**Total Body Surface Area (TBSA) affected^a^**		**Effectiveness^a^**	**Follow-up^a^**	**References**

Cell therapy	Allogeneic hBM-MSCs	Phase I/II Clinical Trial (Single Group Assignment- Open Label)	10 (5/5)	4.8 ± 3.8 (1–11)	Three intravenous infusions of hBM-MSCs on Days 0, 7, and 28, at a dose of 1× 10^6^ to 3 × 10^6^ cells/kg	78% of adverse events were not related to the hBM-MSCs infusions 2 severe events of DMSO odor Mild nausea and abdominal pain and bradycardia were observed during 2 infusions (each)	23.2 ± 11.2%		Mean quality of life score (higher is worse) reported by parents was 41.9 at baseline vs. 39.0 at day 180	180 days	Petrof et al., 2015
	Allogeneic hBM-MSCs	Randomized clinical trial (parallel assignment- double blind)	7 (3/4)	3.8 ± 2 (1–6)	Cyclosporine suspension in a dose of 5 mg/kg per day Intravenous injection of hBM-MSCs (from 70 to 150 × 10^6^ cells/patient)	None	67.1 ± 11.1% (50–80%)		The mean number of new blister formation decreased significantly after treatment from 43 ± 21.2 to 9 ± 10.97 in cyclosporine’s group and from 49 ± 1.8 to 13 ± 8.5 in placebo’s group	1 year	El-Darouti et al., 2016
			7 (3/4)	7.7 ± 6.4 (2–20)	Placebo suspension without cyclosporine Intravenous injection of hBM-MSCs (from 70 to 150 × 10^6^ cells/patient)		64.3 ± 18.1% (40–80%)				
	Allogeneic hBM-MSCs	Phase II Clinical Trial	10 (5/5)	9.1 ± 7 (1.8–22.1)	hBM-MSCs infusions	16% of transplants complicated by veno-occlusive disease of the liver Low rates of acute (0%) and chronic (10%, *n* = 1) graft versus host disease	49.5%		Reduction of surface area of blisters/erosion to 27.5%	1 year	(Ebens et al., 2019) NCT02582775 (MT2015-20: Biochemical Correction of Severe EB by Allo HSCT and Serial Donor MSCs – Full Text View – ClinicalTrials.gov)^3^
	Allogeneic hBM-MSCs	Phase I/II Clinical Trial (Single Group Assignment- Open Label)	10 (5/5)	36.1 ± 9 (26–55)	2 intravenous infusions of hBM-MSCs (24 × 10^6^ to 4 × 10^6^ cells/kg)	None	-		There was a transient reduction in disease activity scores (8/10 subjects) and a significant reduction in itch	1 year	(Rashidghamat et al., 2020) NCT02323789 (Mesenchymal Stromal Cells in Adults With Recessive Dystrophic Epidermolysis Bullosa – Full Text View – ClinicalTrials.gov)^2^
	Allogeneic hUCB-MSCs	Phase I/II Clinical Trial (Single Group Assignment- Open Label)	5	(10–60)	Intravenous injection of 3 doses of 3 × 10^6^ cells/kg	–	−		No results posted	8 months	NCT04520022 (Safety and Effectiveness Study of Allogeneic Umbilical Cord Blood-derived Mesenchymal Stem Cell in Patients With RDEB – Full Text View – ClinicalTrials.gov)^4^
	Allogeneic haploidentical hBM-MSCs	Phase I/II Clinical Trial (Single Group Assignment- Open Label)	9	(1–18)	Intravenous injection of 3 doses of 2× 10^6^ to 3 × 10^6^ cells/kg	−	−		No results posted	5 years	NCT04153630 (Safety Study and Preliminary Efficacy of Infusion Haploidentical Mesenchymal Stem Cells Derived From Bone Marrow for Treating Recessive Dystrophic Epidermolysis Bullosa – Full Text View – ClinicalTrials.gov)^5^
	Allogeneic hMSCs (not defined)	Phase I/IIa Multicenter Clinical Trial (Single Group Assignment- Open Label)	16	Up to 55	Intravenous injection of 3 doses of 2 × 10^6^ cells/kg	–	−		No results posted	24 months	NCT03529877 (Allogeneic ABCB5-positive Stem Cells for Treatment of Epidermolysis Bullosa – Full Text View – ClinicalTrials.gov)^1^

*^*a*^Expression of measures: mean ± standard deviation (range).*

*^1^Allogeneic ABCB5-positive Stem Cells for Treatment of Epidermolysis Bullosa – Full Text View – ClinicalTrials.gov Available at: https://www.clinicaltrials.gov/ct2/show/NCT03529877?term=mesenchymal+stem+cells&recrs=abdefgh&cond=Recessive+Dystrophic+Epidermolysis+Bullosa&strd_s=01%2F01%2F2015&strd_e=12%2F31%2F2020&draw=2&rank=3 [Accessed January 13, 2021].*

*^2^Mesenchymal Stromal Cells in Adults With Recessive Dystrophic Epidermolysis Bullosa – Full Text View – ClinicalTrials.gov Available at: https://clinicaltrials.gov/ct2/show/NCT02323789 [Accessed February 14, 2021].*

*^3^MT2015-20: Biochemical Correction of Severe EB by Allo HSCT and Serial Donor MSCs – Full Text View – ClinicalTrials.gov Available at: https://clinicaltrials.gov/ct2/show/NCT02582775 [Accessed February 14, 2021].*

*^4^Safety and Effectiveness Study of Allogeneic Umbilical Cord Blood-derived Mesenchymal Stem Cell in Patients With RDEB – Full Text View – ClinicalTrials.gov Available at: https://www.clinicaltrials.gov/ct2/show/NCT04520022?term=mesenchymal+stem+cells&recrs=abdefgh&cond=Recessive+Dystrophic+Epidermolysis+Bullosa&strd_s=01%2F01%2F2015&strd_e=12%2F31%2F2020&draw=2&rank=1 [Accessed January 13, 2021].*

*^5^Safety Study and Preliminary Efficacy of Infusion Haploidentical Mesenchymal Stem Cells Derived From Bone Marrow for Treating Recessive Dystrophic Epidermolysis Bullosa – Full Text View – ClinicalTrials.gov Available at: https://www.clinicaltrials.gov/ct2/show/NCT04153630?term=mesenchymal+stem+cells&recrs=abdefgh&cond=Recessive+Dystrophic+Epidermolysis+Bullosa&strd_s=01%2F01%2F2015&strd_e=12%2F31%2F2020&draw=2&rank=2 [Accessed January 13, 2021].*

At preclinical level (Table 5), only two studies have investigated the use of hBM-MSCs (Kühl et al., 2015) or hUCB-MSCs (Petrova et al., 2020). Interestingly, these studies show a different strategy to evaluate the potential effect of advanced therapies for RDEB. In the first case, Kühl et al. (2015) used a hypomorphic mouse model of dystrophic epidermolysis bullosa, meanwhile, Petrova et al. (2020) developed TESS manufactured with RDEB fibroblasts and keratinocytes and compared the results with a TESS fabricated with hUCB-MSCs transduced with *COL7A1* gene.

Kühl et al. (2015) evaluated a cell therapy for 12 weeks where after provoking full thickness skin wounds, 2 × 10^6^ hBM-MSCs were injected at the wound edges at 8 and 56 h after generating the wounds. Results revealed that after 3, 6, and 7 days, the MSC-injected wounds remained significantly smaller than PBS-injected wounds (day 3, 38.1 ± 2.7% vs. 59.3 ± 6.4%; day 6, 30.3 ± 2.4% vs. 39.7 ± 4.1%; and day 7, 21.8 ± 1.6% versus 31.8 ± 4.7%) and moreover, after 12 weeks, healed MSC-injected hypomorphic wounds contained 2.1 ± 0.5 immature anchoring fibrils/μm lamina densa with a thickness of 19.6 ± 1.3 nm which was lower than wild-type skin (3.8 ± 0.3 anchoring fibrils/μm lamina densa with an average maximal thickness of 32.5 ± 1.6 nm), but fibrils were clearly functional and stabilized the skin (Kühl et al., 2015).

Research by Petrova et al. (2020) combined CT, TE and gene therapy (GT) which are the three types of advanced therapies. In this case, full-thickness wounds were generated and then devitalized. Authors manufactured three types of TESSs: (i) RDEB fibroblasts mixed with *COL7A1*-transduced hUCB-MSCs at a 1:1 ratio and primary RDEB keratinocytes seeded on top; (ii) RDEB fibroblasts alone and primary RDEB keratinocytes seeded on top; and (iii) wild type fibroblasts alone and wild type keratinocytes seeded on top. In addition, after 6–8 weeks mice received two intradermal injections of 0.5 × 10^6^ *COL7A1*-transduced hUCB-MSCs or 2 × 10^6^ *COL7A1*-transduced hUCB-MSCs via tail vein (controls without cells were also injected). Results revealed that mice treated with TESSs constituted of hUCB-MSCs had an abundance of sublamina densa fibrillary structures that bore the ultrastructural characteristics of normal anchoring fibril. Evaluating cell therapy, small blisters were seen in the control intradermal-injected animals but not in the animals that received hUCB-MSCs intradermal injection. No evidence were observed that systematically injected hUCB-MSCs migrated to sites of skin grafts.

On balance, the use of hMSCs for the treatment of RDEB it is a promising strategy, however, due to genetic conditions of the disease, the combination of different advanced therapies seems to be the best option which implies more preclinical studies before translating to the clinical environment.

#### Clinical Studies

Since 2015, seven clinical trials have evaluated the use of hMSCs as CT strategy for the treatment of RDEB (Table 5). All of them used allogeneic hMSCs ant the most studied population was hBM-MSCs (5), although hUCB-MSCs (1) and not-defined hMSCs (1) were also analyzed.

Petrof et al. (2015) evaluated the application of three intravenous injections of allogeneic hBM-MSCs (1×10^6^ cells/kg to 3 × 10^6^ cells/kg) in 10 patients (4.8 ± 3.8 years old). They reported that most of the adverse events observed were not related to the hBM-MSCs and after 180 days of follow-up skin biopsies revealed no increase in type VII collagen expression and no new anchoring fibrils formation but quality of life increased and pain decreased.

Similar clinical studies were developed by Ebens et al. (NCT02582775) and Rashidghamat et al. (NCT02323789). In each clinical trial 10 patients received intravenous infusions of hBM-MSCs [2× 10^6^ cells/kg to 4 × 10^6^ cells/kg (Rashidghamat et al., 2020)]. In the case of Ebens et al. two of the transplants provoked veno-occlusive disease of the liver and 1 of the patients developed graft versus host disease (Ebens et al., 2019). After 1 year of follow-up, skin biopsies showed stable (*n* = 7) to improved (*n* = 2) type VII collagen protein expression and gain of anchoring fibril components (*n* = 3) (Ebens et al., 2019) and total blister count over the entire body surface area showed a decrease compared with baseline (Rashidghamat et al., 2020).

El-Darouti et al. (2016) also used hBM-MSCs for the treatment of RDEB but in this case, they compared the intravenous injection of these cells (70 × 10^6^ cells to 150 × 10^6^ cells) with or without a cyclosporine suspension (5 mg/kg) in 14 patients. No adverse events were observed in any of the and the mean number of new blister formation decreased from 43 ± 21.2 to 9 ± 10.97 in cyclosporine’s group and from 49 ± 1.8 to 13 ± 8.5 in the other group. After 1 year, improvement was continuous in two patients from cyclosporine’s group, whereas the remaining patients showed gradual loss of improvement.

Finally, other three clinical trials evaluated the use of allogeneic hUCB-MSCs (NCT04520022), allogeneic haploidentical hBM-MSCs (NCT04153630) and allogeneic hMSCs (NCT03529877). In all cases the route of administration was intravenous injection (3 doses of 2 × 10^6^ cells/kg to 3 × 10^6^ cells/kg), however, no results are posted.

On balance, advanced therapies based on the use of hMSCs for the treatment of RDEB seems to be a promising strategy, although more research is required. Until now, all clinical studies have evaluated the use of these cells as cell therapy, however, at preclinical level, one study has investigated the combination of CT, TE, and GE, but its translation to a clinical environment is still far from achieving. The genetic conditions of the disease and the lack of long-term information about genetic modification of cells are a big handicap. Considering the cell source, hBM-MSCs were the population most used (5/7) and the route of administration preferred was intravenous injection.

### Psoriasis

Psoriasis is a long-term (chronic) inflammatory skin disease with a strong genetic predisposition and autoimmune pathogenic traits that causes erythematous, itchy scaly patches. Worldwide prevalence is about 2% (Christophers, 2001). Five types of psoriasis have been reported: (i) plaque psoriasis or psoriasis vulgaris; (ii) eruptive psoriasis, which is characterized by scaly teardrop-shaped spots; (iii) inverse psoriasis, also called intertriginous or flexural psoriasis that is usually found in folds of skin; (iv) pustular psoriasis; and (v) erythrodermic psoriasis, which is a rare but very serious complication of psoriasis (Boehncke and Schön, 2015).

Due to the etiology of the psoriasis, the anti-inflammatory and immunomodulatory capacities of hMSCs (Di Nicola et al., 2002) have emerged as a useful tool for the development of possible advanced therapies, mainly CT. Their potential has been demonstrated *in vitro*, being capable of restoring the physiological phenotypical profile of psoriatic MSCs (Campanati et al., 2018).

#### Preclinical *in vivo* Studies

Since 2015, five *in vivo* studies have evaluated the potential use of hMSCs as CT for psoriasis (Table 6; Sah et al., 2016; Lee et al., 2017; Kim J.Y. et al., 2018; Chen et al., 2019; Imai et al., 2019). To that purpose, in all cases, researches induced the disease through the topical application of imiquimod (IMQ), except in the case of Yun-Sang et al. (Lee et al., 2017) where psoriasis was induced by intradermal injection of IL-23.

**TABLE 6:** Preclinical *in vivo* studies of hMSCs used as advanced therapy for psoriasis in the last 5 years.

Type of advanced therapy	Cells	Type of hMSC treatment evaluated	Animals	Disease models	Follow-up	References
Cell therapy and gene therapy	hUCB-MSCs	1-hUCB-MSC group: 2 × 10^6^ cells injected subcutaneously 24 h before and at day 6 of imiquimod application 2-hUCB-MSC-transduced group: 2 × 10^6^ cells transduced with extracellular superoxide dismutase (SOD3) and injected subcutaneously 24 h before and at day 6 of imiquimod application 3-Control group: subcutaneous injections of an equal volume of phosphate-buffered saline at the same time points	– C57BL/6 mice	A mouse model of IMQ-induced psoriasis-like inflammation	12 days	Sah et al., 2016
Cell therapy	hUCB-MSCs	1-hUCB-MSC group: 2 × 10^6^ cells were injected subcutaneously on day 1 and 7 after induction of psoriasis-like skin 2-Control group: subcutaneous injections of an equal volume of phosphate-buffered saline at the same time points	– C57/BL6 male mice	IL-23-mediated psoriasis-like skin inflammation mouse model IMQ-induced psoriasis-like skin inflammation mouse model	15 days	Lee et al., 2017
	hPT-MSCs	1-hPT-MSC group: 10^6^ cells via the mouse tail vein on days 1 and 3 of the imiquimod application period 2-Control group: Mice received an intravenous injection of an equal volume of phosphate-buffered saline (PBS) via tail vein at the same time points	– Female C57BL/6 mice	IMQ-induced psoriasis-like skin inflammation mouse model	7 days	Kim J.Y. et al., 2018
	hA-MSCs	1-hA-MSC group: 2 × 10^5^ cells injected intravenously 2-Control group: mouse serum injected intravenously	– B6 mice	A mouse model of IMQ-induced psoriasis-like inflammation	5 days	Imai et al., 2019
	hUCB-MSCs	1-hUCB-MSC group: 1 × 10^5^ cells injected intravenously 2-Control group: phosphate-buffered saline injected intravenously	– Female BALB/c mice	A mouse model of IMQ-induced psoriasis-like inflammation	13 days	Chen et al., 2019

Interestingly, one study combined CT with GT strategy (Sah et al., 2016) where 2 × 10^6^ hUCB-MSCs injected subcutaneously were transduced with extracellular superoxide dismutase (SOD3) demonstrating that enhanced the immunomodulatory and antioxidant activities of hMSCs and therefore, prevented psoriasis development after 12 days of follow-up.

Rest of the reports studied hUCB-MSCs (Lee et al., 2017; Chen et al., 2019), palatine tonsil (hPT-MSCs) (Kim J.Y. et al., 2018) or hA-MSCs (Imai et al., 2019) as CT strategy for 10 ± 4.8 days. Studies which evaluated the use of hUCB-MSCs demonstrated that proinflammatory cytokines such as IL-6, IL-17, and TNF-α were inhibited but IL-10 was significantly increased (Lee et al., 2017; Chen et al., 2019). Lee et al. (2017) injected subcutaneously 2 × 10^6^ cells on days 1 and 7 after induction of psoriasis-like skin meanwhile, Chen et al. (2019) injected intravenously 1 × 10^5^ cells, but in both cases, hUCB-MSCs did not only prevent but also treat psoriasis-like skin (Lee et al., 2017; Chen et al., 2019).

In the case where hPT-MSCs (10^6^ cells) and hA-MSCs (2 × 10^5^ cells) were applied, intravenous injection was the route of administration chosen and, in both cases, suppressed the development of psoriasis (Kim J.Y. et al., 2018; Imai et al., 2019).

On balance, results using hMSCs were better than control groups in all cases and two studies used the Psoriasis Area and Severity Index (PASI); demonstrating in both cases that after 7 to 8 days, PASI values were lower for hMSCs-treated groups (4.5) than non-treated groups (6.5) (Kim J.Y. et al., 2018; Chen et al., 2019).

#### Clinical Studies

Over the past 5 years, nine clinical studies have reported the use of hMSCs for the treatment of different types of psoriasis: psoriasis vulgaris (Chen et al., 2016; De Jesus et al., 2016) (NCT03765957), psoriatic arthritis (De Jesus et al., 2016), moderate to severe psoriasis (Comella et al., 2018) (NCT03265613, NCT03392311, and NCT04275024) and plaque psoriasis (Wang et al., 2020) (NCT02918123) (Table 7).

**TABLE 7:** Clinical studies of hMSCs used as advanced therapy for psoriasis in the last 5 years.

Type of advanced therapy	Cells	Type of clinical study	N (male/female)	Age (years)^a^	hMSC treatment	Safety (Treatment-related adverse events)	Indication	Effectiveness^a^	Follow-up^a^	References
Cell therapy	Allogeneic hUCB-MSCs	Case report	2 (1/1)	30.5 ± 6.4	1–3 infusions of hUCB-MSCs (10^6^ cells/kg)	None	Psoriasis vulgaris	No symptoms of psoriatic relapse were observed	4–5 years	Chen et al., 2016
	Autologous hAT-MSCs	Case report	2 (1/1)	43 ± 21.2	2–3 intravenous infusions of hAT MSCs at a dose of 0.5 –3.1 × 10^6^ cells/kg	None	Psoriasis vulgaris (PV) and psoriatic arthritis (PA)	PASI changed from 21 to 9 and from 24 to 8.3	0.63 years	De Jesus et al., 2016
	Autologous hAT-MSCs	Case report	1 (1/0)	43	Intravenous injection of 3 – 6 × 10^7^ hAT-MSCs in normal saline	None	Severe psoriasis	PASI score decreased from 50.4 to 0.3	1 year	Comella et al., 2018
	Allogeneic hG-MSCs	Case report	1 (1/0)	19	5 bolus injections of hG-MSCs (3 × 10^6^/kg/infusion)	None	Plaque psoriasis	After 3 years the disease was resolved	3 years	Wang et al., 2020
	Allogeneic hUCB-MSCs	Early Phase I clinical trial (Single Group Assignment- Open Label)	12	(18–65)	Different doses of hUCB-MSCs from 1.5 × 10^6^ to 3 × 10^6^	–	Psoriasis vulgaris	–	6 months	NCT03765957 (Clinical Research on Treatment of Psoriasis by Human Umbilical Cord-derived Mesenchymal Stem Cells – Full Text View – ClinicalTrials.gov)^1^
	Allogeneic hAT-MSCs	Phase I/II Clinical Trial (Single Group Assignment- Open Label)	7	(18–65)	Intravenous injection of 5 × 10^5^ cells/kg at week 0, week 4 and week 8	–	Moderate to severe psoriasis	–	12 weeks	NCT03265613 (Safety and Efficacy of Expanded Allogeneic AD-MSCs in Patients With Moderate to Severe Psoriasis – Full Text View – ClinicalTrials.gov)^4^
	Allogeneic hAT-MSCs	Phase I/II Clinical Trial (Single Group Assignment- Open Label)	5		Intravenous injection of 2 × 10^6^ cells/kg at week 0, week 2, week 4, week 6 and week 8. In addition, calcipotriol ointment was topically applied twice daily	–	Moderate to severe psoriasis	–	12 weeks	NCT03392311 (Efficacy and Safety of AD-MSCs Plus Calpocitriol Ointment in Patients With Moderate to Severe Psoriasis – Full Text View – ClinicalTrials.gov)^3^
	Allogeneic hAT-MSCs	Clinical Trial (Single Group Assignment- Open Label)	8	(18–65)	Intravenous injection of 2 × 10^6^ cells/kg at week 0, week 2, week 4, week 6 and week 8. In addition, oral PSORI-CM01 Granule plus calcipotriol ointment was topically applied twice daily	–	Moderate to severe psoriasis	–	12 weeks	NCT04275024 (Efficacy and Safety of AD-MSCs Plus Calpocitriol Ointment and PSORI-CM01 Granule in Psoriasis Patients – Full Text View – ClinicalTrials.gov)^2^
	Allogeneic hUCB-MSCs	Phase I Clinical Trial (Single Group Assignment- Open Label)	9	(19–65)	Different doses of hUCB-MSCs from 5 × 10^7^ to 2 × 10^8^, subcutaneously injected	–	Moderate to severe plaque psoriasis	–	144 weeks	NCT02918123 (Safety of FURESTEM-CD Inj. in Patients With Moderate to Severe Plaque-type Psoriasis – Full Text View – ClinicalTrials.gov)^5^

*^*a*^Expression of measures: mean ± standard deviation (range).*

*^1^Clinical Research on Treatment of Psoriasis by Human Umbilical Cord-derived Mesenchymal Stem Cells – Full Text View – ClinicalTrials.gov Available at: https://www.clinicaltrials.gov/ct2/show/NCT03765957?term=mesenchymal+stem+cells&recrs=abdefgh&cond=Psoriasis&strd_s=01%2F01%2F2015&strd_e=12%2F31%2F2020&draw=2&rank=1 [Accessed January 13, 2021].*

*^2^Efficacy and Safety of AD-MSCs Plus Calpocitriol Ointment and PSORI-CM01 Granule in Psoriasis Patients – Full Text View – ClinicalTrials.gov Available at: https://www.clinicaltrials.gov/ct2/show/NCT04275024?term=mesenchymal+stem+cells&recrs=abdefgh&cond=Psoriasis&strd_s=01%2F01%2F2015&strd_e=12%2F31%2F2020&draw=1&rank=4 [Accessed January 21, 2021].*

*^3^Efficacy and Safety of AD-MSCs Plus Calpocitriol Ointment in Patients With Moderate to Severe Psoriasis – Full Text View – ClinicalTrials.gov Available at: https://www.clinicaltrials.gov/ct2/show/NCT03392311?term=mesenchymal+stem+cells&recrs=abdefgh&cond=Psoriasis&strd_s=01%2F01%2F2015&strd_e=12%2F31%2F2020&draw=1&rank=3 [Accessed January 21, 2021].*

*^4^Safety and Efficacy of Expanded Allogeneic AD-MSCs in Patients With Moderate to Severe Psoriasis – Full Text View – ClinicalTrials.gov Available at: https://www.clinicaltrials.gov/ct2/show/NCT03265613?term=mesenchymal+stem+cells&recrs=abdefgh&cond=Psoriasis&strd_s=01%2F01%2F2015&strd_e=12%2F31%2F2020&draw=2&rank=2 [Accessed January 21, 2021].*

*^5^Safety of FURESTEM-CD Inj. in Patients With Moderate to Severe Plaque-type Psoriasis – Full Text View – ClinicalTrials.gov Available at: https://www.clinicaltrials.gov/ct2/show/NCT02918123?term=mesenchymal+stem+cells&recrs=abdefgh&cond=Psoriasis&strd_s=01%2F01%2F2015&strd_e=12%2F31%2F2020&draw=1&rank=5 [Accessed January 21, 2021].*

All studies applied CT strategies, two of them used autologous hAT-MSCs (De Jesus et al., 2016; Comella et al., 2018) and the others, evaluated the use of allogeneic cells from different sources: hUCB-MSCs (Chen et al., 2016) (NCT03765957, NCT02918123), hAT-MSCs (NCT03265613, NCT03392311, NCT04275024), and gingival mucosa MSCs (hG-MSCs) (Wang et al., 2020).

Results were reported in the four case reports analyzed (Chen et al., 2016; De Jesus et al., 2016; Comella et al., 2018; Wang et al., 2020) and despite of the fact that different hMSCs populations were used, adverse events were not observed in none of them.

Chen et al. (2016) evaluated the use of allogeneic hUCB-MSCs by 1–3 infusions (doses of 10^6^ cells/kg) in 2 patients with psoriasis vulgaris. After a follow-up of 4–5 years no symptoms of psoriatic relapse were observed. Something similar occurred in the study of Wang et al. (2020) were hG-MSCs were administered by bolus injections (5 doses of 3 × 10^6^/kg) in a patient with plaque psoriasis, demonstrating that after 3 years the disease was resolved.

In contrast, De Jesus et al. (2016) and Comella et al. (2018) applied autologous hAT-MSCs injected intravenously [2–3 doses of 0.5–3.1 × 10^6^ cells/kg or a unique dose of 6 × 10^7^ cells]. In the first case, significant improvement was noted in lesions of 2 patients with psoriasis vulgaris and psoriatic arthritis (PASI changed from 21 to 9 and from 24 to 8.3, respectively) and joint pain was reduced, meanwhile, in the second case, psoriasis area and severity PASI score decreased from 50.4 at baseline to 0.3 at 1-month follow-up (Comella et al., 2018).

Rest of studies reviewed were clinical trials, but no results have been posted yet. In these, allogeneic hAT-MSCs (3) or hUCB-MSCs (2) alone (NCT03765957, NCT03265613, and NCT02918123) or in combination with other treatments such as calcipotriol ointment (NCT033923119) or oral PSORI-CM01 Granule (NCT04275024) has been evaluated. Type of treatments varied from doses of 5 × 10^5^ cells/kg to a unique injection of 2 × 10^8^ cells and the preferred source of administration was intravenously although one study analyzed the application of subcutaneous injections (NCT02918123).

According to the investigations reviewed, at preclinical and clinical level, for the treatment of different types of psoriasis the best advanced therapy based on the use of hMSCs is cell therapy. Results in most of the cases demonstrated a decrease of the PASI values and in some patients, psoriasis disappeared. Considering the route of administration, intravenous injections are the most analyzed and the most promising hMSC populations are hAT-MSCs and hUCB-MSCs, although more research is required to standardize the dose and determine the autologous or allogeneic nature of the cells.

### Atopic Dermatitis

Atopic dermatitis is a chronic inflammatory pruritic skin disease that affects a large number of children and adults (Akdis et al., 2006), although it can improve significantly, or even clear completely, in some children as they get older. The exact cause of atopic eczema is unknown, but the imbalance of Th2 to Th1 cytokines plays a crucial role (David Boothe et al., 2017).

#### Preclinical *in vivo* Studies

In the last 5 years, seven *in vivo* studies have reported the use of hMSCs as advanced therapy (Table 8; Kim et al., 2015, Kim J.M. et al., 2018; Shin et al., 2017; Sah et al., 2018; Lee et al., 2019; Park et al., 2019, Park H.H. et al., 2020). The disease model preferred to induce atopic dermatitis was the use of *Dermatophagoides farina* extract alone (Kim et al., 2015; Shin et al., 2017) or combined with other agents such as house dust mites (Park H.H. et al., 2020) and 4% sodium dodecyl sulfate (SDS) (Lee et al., 2019). Other substances used were ovalbumin (Sah et al., 2018), acetone and 2, 4-dinitrochlorobenzene (DNCB): olive oil mixture (4:1 vol/vol) (Kim J.M. et al., 2018) and *Aspergillus fumigatus* extract (Park et al., 2019).

**TABLE 8:** Preclinical *in vivo* and clinical studies of hMSCs used as advanced therapy for atopic dermatitis in the last 5 years.

PRECLINICAL IN VIVO STUDIES											

Type of advanced therapy	Cells		Type of hMSC treatment evaluated						Disease models	Follow-up	References
Cell therapy	hUCB-MSCs		1-hUCB-MSCs group: 2 × 10^6^ cells injected intravenously on days 2, 9, 16 and 23. 2-Muramyl dipeptide – hUCB-MSCs (intravenous) group: 2 × 10^6^ injected intravenously on days 2, 9, 16 and 23 previous administration of muramyl dipeptide. 3-Muramyl dipeptide – hUCB-MSCs (subcutaneous) group: 2 × 10^6^ injected subcutaneously on days 2, 9, 16 and 23 previous administration of muramyl dipeptide.						*Dermatophagoides farinae*-induced murine atopic dermatitis model	30 days	Kim et al., 2015
	hAT-MSCs		1-hAT-MSCs (low) group: 2 × 10^5^ cells injected intravenously 2-hAT-MSCs (high) group: 2 × 10^6^ cells injected intravenously 3-Fibroblasts group: Human dermal fibroblasts injected intravenously						*Dermatophagoides farinae*-induced murine atopic dermatitis model	14 days	Shin et al., 2017
	hAT-MSCs		1-hAT-MSCs group: 10^6^ cells injected intravenously on days 0 and 11 2-Fibroblasts group: Human dermal fibroblasts injected intravenously						Acetone and 2, 4-dinitrochlorobenzene (DNCB): olive oil mixture (4:1 vol/vol) induced atopic dermatitis	24 days	Kim J.M. et al., 2018
	hUCB-MSCs		1-hUCB-MSCs group: subcutaneous administration of 10^6^ cells 2-MC-hUCB-MSCs primed group: subcutaneous administration of 10^6^ cells primed with mast cells granules 3-Fibroblasts group: subcutaneous administration of 10^6^ cells						4% sodium dodecyl sulfate (SDS) and *Dermatophagoides farinae* extract induced atopic dermatitis	14 days	Lee et al., 2019
	hWJ-MSCs		1-hWJ-MSCs group: subcutaneous administration of 2 × 10^6^ cells 2-hWJ-MSCs-poly I:C group: subcutaneous administration of 2 × 10^6^ cells primed with poly I:C 3-hWJ-MSCs- IFN-γ group: subcutaneous administration of 2 × 10^6^ cells primed with IFN-γ						*Aspergillus fumigatus* extract to induce atopic dermatitis	5 days	Park et al., 2019
Cell therapy and gene therapy	hUCB-MSCs		1-hUCB-MSCs group: 2 × 10^6^ cells injected subcutaneous on day 20, 28 and 42 and one time on day 56 after the development of atopic dermatitis 2-hUCB-MSCs transduced group: 2 × 10^6^ superoxide dismutase 3 (SOD3) transduced cells injected subcutaneous on day 20, 28 and 42 and one time on day 56 after the development of atopic dermatitis						Ovalbumin – atopic dermatitis induced mouse model	56 days	Sah et al., 2018
	hUCB-MSCs		1-hUCB-MSCs group: subcutaneous injection of 2 × 10^6^ cells 2-hUCB-MSCs transfected control group: subcutaneous injection of 2 × 10^6^ cells transfected with siRNA control 3-hUCB-MSCs transfected siTGF-β group: subcutaneous injection of 2 × 10^6^ cells transfected with siRNA targeting TGF-β						*Dermatophagoides farina* extract containing components of house dust mites to induce atopic dermatitis	7 days	Park H.H. et al., 2020

**CLINICAL STUDIES**											

**Type of advanced therapy**	**Cells**	**Type of clinical study**	**N (male/female)**	**Age (years)^a^**	**hMSC treatment**	**Safety (Treatment-related adverse events)**	**Indication**	**Effectiveness^a^**	**Follow-up^a^**	**References**	

Cell therapy	Allogeneic hUCB-MSCs	Phase I/II Randomized Clinical Trial (Double Blind)	For safety:33 For efficacy: 25 (16/5)	For safety: 20–60 For efficacy: 28.63 (20–60)	Subcutaneous administration of hUCB-MSCs at two different doses (low dose; 2.5 × 10^7^ cells or high dose; 5.0 × 10^7^ cells)	Low dose: 12% of patients (1 event per patient) High dose: 56% patients (1.78 events per patient)	Moderate to severe Atopic Dermatitis (Severity Scoring for Atopic Dermatitis (SCORAD) > 20)	After 12 weeks, SCORAD value was reduced: Low dose: −28.04 ± 6.20% High dose: −49.97 ± 4.33%	12 weeks	Kim et al., 2017	
	Autologous hMSCs (not defined)	Phase I Clinical Trial (Single Group Assignment- Open Label)	13	(19–70)	Two different doses of hMSCs administered by intravenous infusion: 1 × 10^8^ cells or 3 × 10^8^ cells	–	Moderate to Severe, Subacute and Chronic Atopic Dermatitis	–	12 weeks	NCT02888704 (Safety and Efficacy of ADSTEM Inj. in Patients With Moderately Subacute and Chronic Atopic Dermatitis – Full Text View – ClinicalTrials.gov)^2^	
	Autologous hMSCs (not defined)	Observational Study	11	(19–70)	Two different doses of hMSCs administered by intravenous infusion: 1 × 10^8^ cells or 3 × 10^8^ cells	–	Moderate to Severe, Subacute and Chronic Atopic Dermatitis	–	60 months	NCT03252340 (Safety of ADSTEM Injection in Patients With Moderate to Severe Subacute and Chronic Atopic Dermatitis – Full Text View – ClinicalTrials.gov)^4^	
	Allogeneic hBM-MSCs	Phase I/II Multicenter Randomized Clinical Trial (Parallel Assignment- Quadruple Blind)	92	Older than 19	Three doses of 10^6^ cells injected intravenously at weeks 0, 2 and 4	–	Moderate to Severe Atopic Dermatitis	–	24 weeks	NCT04179760 (Safety and Efficacy of SCM-AGH in Subjects With Moderate to Severe Atopic Dermatitis – Full Text View – ClinicalTrials. gov)^3^	
	hAT-MSCs	Phase II Multicenter Randomized Clinical Trial (Parallel Assignment- Double Blind)	118	(19–70)	Two intravenous injections of 0.5 × 10^8^ cells	–	Moderate to Severe, Subacute and Chronic Atopic Dermatitis	–	16 weeks	NCT04137562 (Safety and Efficacy in Patients With Moderate to Severe Subacute and Chronic Atopic Dermatitis – Full Text View – ClinicalTrials. gov)^1^	

*^*a*^Expression of measures: mean ± standard deviation (range).*

*^1^Safety and Efficacy in Patients With Moderate to Severe Subacute and Chronic Atopic Dermatitis – Full Text View – ClinicalTrials.gov Available at: https://www.clinicaltrials.gov/ct2/show/NCT04137562?term=mesenchymal+stem+cells&recrs=abdefgh&cond=atopic+dermatitis&strd_s=01%2F01%2F2015&strd_e=12%2F31%2F2020&draw=2&rank=4 [Accessed January 21, 2021].*

*^2^Safety and Efficacy of ADSTEM Inj. in Patients With Moderately Subacute and Chronic Atopic Dermatitis – Full Text View – ClinicalTrials.gov Available at: https://www.clinicaltrials.gov/ct2/show/study/NCT02888704?term=mesenchymal+stem+cells&recrs=abdefgh&cond=atopic+dermatitis&strd_s=01%2F01%2F2015&strd_e=12%2F31%2F2020&draw=2&rank=1 [Accessed January 21, 2021].*

*^3^Safety and Efficacy of SCM-AGH in Subjects With Moderate to Severe Atopic Dermatitis – Full Text View – ClinicalTrials.gov Available at: https://www.clinicaltrials.gov/ct2/show/NCT04179760?term=mesenchymal+stem+cells&recrs=abdefgh&cond=atopic+dermatitis&strd_s=01%2F01%2F2015&strd_e=12%2F31%2F2020&draw=2&rank=3 [Accessed January 21, 2021].*

*^4^Safety of ADSTEM Injection in Patients With Moderate to Severe Subacute and Chronic Atopic Dermatitis – Full Text View – ClinicalTrials.gov Available at: https://www.clinicaltrials.gov/ct2/show/NCT03252340?term=mesenchymal+stem+cells&recrs=abdefgh&cond=atopic+dermatitis&strd_s=01%2F01%2F2015&strd_e=12%2F31%2F2020&draw=2&rank=2 [Accessed January 21, 2021].*

##### Cell Therapy

Among studies reviewed, five reported the use of hMSCs as CT (Table 8; Kim et al., 2015, Kim J.M. et al., 2018; Shin et al., 2017; Lee et al., 2019; Park et al., 2019). Mean follow-up of these studies was 14.4 ± 9.7 days and hMSCs analyzed were hUCB-MSCs (Kim et al., 2015; Lee et al., 2019), hAT-MSCs (Shin et al., 2017; Kim J.M. et al., 2018), and hWJ-MSCs (Park et al., 2019).

Kim et al. (2015) compared two administration routes: on the one hand, injected 2 × 10^6^ hUCB-MSCs intravenously only or combined with a pretreatment of muramyl dipeptide. On the other hand, also evaluated the same number of cells injected subcutaneously combined with pretreatment of muramyl dipeptide. In all cases, mice were treated on days 2, 9, 16, and 23 and results were compared with untreated and healthy mice. Results indicated that subcutaneous administration of hUCB-MSCs and muramyl dipeptide exhibited prominent protective effects against atopic dermatitis, and suppressed the infiltration and degranulation of mast cells (Kim et al., 2015). In a similar way, Lee et al. (2019) explored the priming of hUCB-MSCs with mast cells granules as potential strategy of treatment. To that purpose they injected subcutaneously 10^6^ hUCB-MSCs primed or not with these granules reporting that preconditioning with mast cells granules enhanced the therapeutic potential of hUCB-MSCs against atopic dermatitis by improving the immunosuppression and tissue regenerative capacity of the cells and after 14 days, decreased the wound area by 20% more than that achieved with naïve MSCs (Lee et al., 2019).

Regarding to the use of hAT-MSCs Shin et al. (2017) and Kim J.M. et al. (2018) developed similar experiments where different doses of hAT-MSCs were injected intravenously and this therapy was compared with the injection of human dermal fibroblasts. In the first study, the amount of cells injected were 2 × 10^5^ and 2 × 10^6^ demonstrating that administration of a high dose of hAT-MSCs reduced the gross and histological signatures of atopic dermatitis, as well as serum IgE level, mainly (Shin et al., 2017). In the second case, two doses of 10^6^ cells were injected intravenously on days 0 and 11 revealing that hAT-MSCs attenuated clinical symptoms associated with atopic dermatitis, decreased numbers of degranulated mast cells (MCs), IgE level, amount of histamine released, and prostaglandin E2 level (Kim J.M. et al., 2018).

Finally, Park et al. (2019) also evaluated the priming of hWJ-MSCs for the treatment of atopic dermatitis. They compared the subcutaneous administration of 2 × 10^6^ cells alone, 2 × 10^6^ cells primed with poly I:C and 2 × 10^6^ cells primed with IFN-γ. Results indicated that priming with poly I:C or IFN-γ affected the immunomodulatory functions of hWJ-MSCs and enhanced their therapeutic effects and alleviated the features of atopic dermatitis including the clinical symptom score, transepidermal water loss and epidermal thickness.

##### Combined advanced therapy: cell therapy and gene therapy

In addition to the previous studies, other two used a combination of CT and GT (Sah et al., 2018; Park H.H. et al., 2020; Table 8), where 2 × 10^6^ hUCB-MSCs transduced with SOD3 (Sah et al., 2018) or transfected with siRNA targeting TGF-β (Park H.H. et al., 2020) were explored. In the first case (Sah et al., 2018), transduced hUCB-MSCs treated mice showed stronger inhibition of atopic dermatitis phenotype and epidermal hyperplasia and dermal infiltration of mononuclear cells were decreased, but in the case of cells transfected with siRNA targeting TGF-β (Park H.H. et al., 2020), therapeutic effect was lower than in the case of non-transfected cells which indicated that the role of TGF-β is important for atopic dermatitis treatment.

##### Brief conclusion

On balance, the use of hMSCs for the treatment of atopic dermatitis is well study at preclinical level. The main advanced therapy strategy applied is cell therapy and the most analyzed population of hMSCs used is hUCB-MSCs, however, the application of gene therapy will require more safety analysis in order to apply it into a clinical environment.

#### Clinical Studies

In the case of clinical research, five studies have reported the use of hMSCs as advanced therapy for the treatment of moderate to severe atopic dermatitis (Table 8). All applied cell therapy strategy, however, only one has published results (Kim et al., 2017), in contrast to the rest of clinical trials or observational studies reviewed (NCT02888704, NCT03252340, NCT04179760, and NCT04137562).

Allogeneic hMSCs were used in two of the cases (Kim et al., 2017) (NCT04179760) and autologous cells in other two (NCT02888704 and NCT03252340). In one of the clinical trials, information about hMSCs’ donor was not indicated (NCT04137562).

Regarding to the tissue source of different hMSC populations analyzed; hUCB-MSCs, hBM-MSCs and hAT-MSCs were used in one study each ((Kim et al., 2017), NCT04179760 and NCT04137562), but in two cases (interventional and observational studies), origin was not indicated (NCT02888704, NCT03252340).

Kim et al. (2017) evaluated safety and efficacy in 33 patients which received subcutaneous injections of allogeneic hUCB-MSCs at two different doses (low dose; 2.5 × 10^7^ cells or high dose; 5.0 × 10^7^ cells). Results revealed that adverse events such as skin infections, gastrointestinal disorders or general disorders and administration site conditions were observed in 12% of patients which received low dose of hUCB-MSCs, in contrast to, 56% patients who received the high dose. In terms of effectiveness, after 12 weeks, Severity Scoring for Atopic Dermatitis (SCORAD) value was reduced in both groups (low dose: −28.04 ± 6.20%; high dose: −49.97 ± 4.33%), demonstrating that single administration of hUCB-MSCs improved atopic dermatitis condition (Kim et al., 2017).

Considering the remaining studies, they compared the intravenous injection of hMSCs; at different doses (1 × 10^8^ cells or 3 × 10^8^ cells – NCT02888704), different time points (three doses of 10^6^ cells at weeks 0, 2 and 4 – NCT04179760) or to evaluate the single administration of two injections of 0.5 × 10^8^ cells (NCT04137562).

In contrast to preclinical level, the number of clinical studies with published results is limited and there is not enough information to stablish a standardized cell therapy based on the use of hMSCs. However, promising results in animal models and the incremental development of clinical trials could be beneficial for the future treatment of atopic dermatitis.

### Scleroderma

Scleroderma is a collective term covering a range of autoimmune inflammatory conditions in which skin thickening (fibrosis) or sclerosis is a hallmark feature (Orteu et al., 2020) due to the excess production of collagen by the cells located in the connective tissue. There are different subsets of the disease, including localized scleroderma, limited cutaneous systemic sclerosis, diffuse cutaneous systemic sclerosis, and systemic sclerosis sine scleroderma (Fett, 2013).

#### Preclinical *in vivo* Studies

Since 2015, only one preclinical study has investigated the use of hMSCs as advanced therapy for the treatment of scleroderma (Maria et al., 2016; Table 9). In this case, the use of hBM-MSCs and hAT-MSCs as CT was evaluated for the treatment of systemic sclerosis (SSc) induced in mice. Results revealed that the application of 2.5 × 10^6^ hAT-MSCs injected intravenously reported a significant reduction of skin thickness 21 days after treatment and better results than hBM-MSCs application, in terms of skin thickness reduction and extracellular matrix deposition (Maria et al., 2016).

**TABLE 9:** Preclinical *in vivo* and clinical studies of hMSCs used as advanced therapy for scleroderma in the last 5 years.

PRECLINICAL IN VIVO STUDIES										

Type of advanced therapy	Cells		Type of hMSC treatment evaluated					Disease models	Follow-up	References
Cell therapy	hBM-MSCs and hAT-MSCs		1-hBM-MSCs group: intravenous infusion of 2.5 × 10^5^ cells 2-hAT-MSCs group: intravenous infusion of 2.5 × 10^5^ cells 3-Control group: no-treatment of systemic sclerosis induced mice					Daily intradermal injections of hypochlorite (HOCl) to induce systemic sclerosis (SSc) for 21 days	21 days	Maria et al., 2016

**CLINICAL STUDIES**										

**Type of advanced therapy**	**Cells**	**Type of clinical study**	**N (male/female)**	**Age (years)^a^**	**hMSC treatment**	**Safety (Treatment-related adverse events)**	**Indication**	**Effectiveness^a^**	**Follow-up^a^**	**References**

Cell therapy	Autologous hAT-MSCs	Phase I Clinical Trial (Single Group Assignment- Open Label)	12 (0/12) 11 patients with skin fibrosis (sclerodactyly)	54.5 ± 10.3 (34.0–68.0)	3.76 ± 1.85 (1.19–7.07)x10^6^ cells were subcutaneously injected into each lateral side of digits	No serious adverse events occurred during follow-up.	Systemic sclerosis (SSc)	After 12 months a significant improvement of 46.8% for the Scleroderma Health Assessment Questionnaire, of 51.3% for the Cochin Hand Function Scale score and of 63.2% for the Raynaud’s condition score was observed	6 and 12 months	Granel et al., 2015; Guillaume-Jugnot et al., 2015 NCT01813279 (Assessment of the Subcutaneous Reinjection of Human Autologous Adipose-derived Stromal Vascular Fraction (Celution^®^ System) in the Hands of Patients Suffering From Systemic Sclerosis – Full Text View – ClinicalTrials.gov)^3^
	Allogeneic hUCB-MSCs	Case report	2 (0/2)	29.0 (28.0–30.0)	2 × 10^6^ cells/kg, infused intravenously – 2 treatments	None	Systemic sclerosis (SSc)	After one year of treatment, European Scleroderma Study Group activity index was reduced from 9.5 to 3.5 and 1.5, respectively	14 months (12–16)	Wehbe et al., 2016
	Allogeneic hUCB-MSCs	Phase I/II Clinical Trial (Single Group Assignment- Open Label)	14 (3/11)	37.4 ± 14.2 (19.0–67.0)	Three repeated plasmapheresis treatments on days 1, 3 and 5. On day 8, single hUCB-MSC infusion was injected (10^6^ cells/kg)	Adverse events noted were upper respiratory tract infections reported by five patients and diarrhea reported by one patient during follow-up visits	Systemic sclerosis (SSc)	After 12 months of treatment, the mean Modified Rodnan skin score improved from 20.1 ± 3.1 to 13.8 ± 10.2	15.6 ± 4.3 months (7–21)	Zhang et al., 2017 NCT00962923 (Allogeneic Mesenchymal Stem Cells Transplantation for Systemic Sclerosis (SSc) – Full Text View – ClinicalTrials.gov)^2^
	Autologous hAT-MSCs	Case report	1 (0/1)	62.0	Subcutaneous injection of 2.72 × 10^6^ cells	None	Systemic sclerosis (SSc)	No need to further amputation was demonstrated.	3 weeks	Song et al., 2017
	Autologous hAT-MSCs	Case report	6 (2/4)	9.3 ± 7.1 (3.0–20.0)	Co-injection of PRP and hAT-MSCs	None	Cutaneous systemic sclerosis (dcSSc)	Skin elasticity was increased (16.64% for the lip and the 17.80% for the cheek)	3 months	Virzì et al., 2017
	Allogeneic hBM-MSCs	Phase I/II Randomized Clinical Trial (Parallel Assignment- Quadruple Blind)	10	–	Intramuscular injections with 50 × 10^6^ cells	–	Systemic sclerosis (SSc) – Digital ulcers (DUs)	–	12 months	(Van Rhijn-Brouwer et al., 2018) NCT03211793
	Autologous hAT-MSCs	Clinical Trial (Single Group Assignment- Open Label)	7	Older than 18	Subcutaneous injection of hAT-MSCs	–	Systemic sclerosis (SSc) – Digital ulcers (DUs)	–	12 weeks	NCT02975960 (ADMSCs for the Treatment of Systemic Sclerosis – Full Text View – ClinicalTrials.gov)^1^
	Allogeneic hUCB-MSCs	Phase I/II Randomized Clinical Trial (Parallel Assignment- Quadruple-Blind)	18	Older than 18	One or two infusions of 10^6^ cells/kg at Month 0 and (in the case of two infusions) Month 3	–	Systemic scleroderma	–	12 months	NCT04356287 (Treatment With Human Umbilical Cord-derived Mesenchymal Stromal Cells in Systemic Sclerosis – Full Text View – ClinicalTrials.gov)^5^
	Autologous hAT-MSCs	Early Phase I Clinical Trial (Single Group Assignment- Open Label)	20 but only 18 completed the study (3/15)	47 (42–57)	Subcutaneous injection of 3.61 (± 4.34) x10^6^ hAT-MSCs	Three patients reported transient pallor in fingers which was resolved within 10 min after resting and warming	Systemic sclerosis (SSc) – Digital ulcers (DUs)	Raynaud’s Condition Scale presented slight improvement, but with no significance (from 5 to 6)	24 weeks	Park Y. et al., 2020 NCT03060551 (Injection of Autologous Adipose-derived Stromal Vascular Fraction in the Finger of Systemic Sclerosis Patients – Full Text View – ClinicalTrials.gov)^4^

*^*a*^Expression of measures: mean ± standard deviation (range).*

*^1^ADMSCs for the Treatment of Systemic Sclerosis – Full Text View – ClinicalTrials.gov Available at: https://www.clinicaltrials.gov/ct2/show/NCT02975960?term=mesenchymal+stem+cells&recrs=abdefgh&cond=Scleroderma&strd_s=01%2F01%2F2015&strd_e=12%2F31%2F2020&draw=2&rank=1 [Accessed January 21, 2021].*

*^2^Allogeneic Mesenchymal Stem Cells Transplantation for Systemic Sclerosis (SSc) – Full Text View – ClinicalTrials.gov Available at: https://clinicaltrials.gov/ct2/show/NCT00962923 [Accessed February 14, 2021].*

*^3^Assessment of the Subcutaneous Reinjection of Human Autologous Adipose-derived Stromal Vascular Fraction (Celution§System) in the Hands of Patients Suffering From Systemic Sclerosis – Full Text View – ClinicalTrials.gov Available at: https://clinicaltrials.gov/ct2/show/NCT01813279 [Accessed February 14, 2021].*

*^4^Injection of Autologous Adipose-derived Stromal Vascular Fraction in the Finger of Systemic Sclerosis Patients – Full Text View – ClinicalTrials.gov Available at: https://clinicaltrials.gov/ct2/show/NCT03060551 [Accessed February 14, 2021].*

*^5^Treatment With Human Umbilical Cord-derived Mesenchymal Stromal Cells in Systemic Sclerosis – Full Text View – ClinicalTrials.gov Available at: https://www.clinicaltrials.gov/ct2/show/NCT04356287?term=mesenchymal+stem+cells&recrs=abdefgh&cond=Scleroderma&strd_s=01%2F01%2F2015&strd_e=12%2F31%2F2020&draw=2&rank=2 [Accessed January 21, 2021].*

#### Clinical Studies

Ten clinical studies have reported the use of hMSCs as advanced therapy for the treatment of systemic sclerosis in the last 5 years (Table 9; Granel et al., 2015; Guillaume-Jugnot et al., 2015; Wehbe et al., 2016; Song et al., 2017; Virzì et al., 2017; Zhang et al., 2017; Van Rhijn-Brouwer et al., 2018; Park Y. et al., 2020) (NCT02975960 and NCT04356287). Among these studies, three studies were case reports (Wehbe et al., 2016; Song et al., 2017; Virzì et al., 2017), in contrast to the rest which were clinical trials.

Human MSCs used as CT were isolated from adipose tissue (hAT-MSCs) (Granel et al., 2015; Guillaume-Jugnot et al., 2015; Song et al., 2017; Virzì et al., 2017; Park Y. et al., 2020) (NCT02975960), umbilical cord blood (hUCB-MSCs) (Wehbe et al., 2016; Zhang et al., 2017) (NCT04356287), and bone marrow (hBM-MSCs) (Van Rhijn-Brouwer et al., 2018). Interestingly, in all cases where hAT-MSCs were used, autologous source was preferred. However, when hUCB-MSCs or hBM-MSCs, the origin was from allogeneic samples. Administration routes applied were subcutaneous for hAT-MSCs, intravenous for hUCB-MSCs and intramuscular for hBM-MSCs.

Among those studies which applied autologous hAT-MSCs (*n* = 44), the number of cells injected varied from 3.76 ± 1.85 (1.19–7.07) × 10^6^ (Granel et al., 2015; Guillaume-Jugnot et al., 2015), 2.72 × 10^6^ cells (Song et al., 2017) to 3.61 ± 4.34 × 10^6^ (Park Y. et al., 2020). Interestingly, in one of the cases cells were co-injected with PRP (Virzì et al., 2017). None adverse events were observed, except in two cases where transient paresthesia, pains or pallor in hands were observed (Granel et al., 2015; Guillaume-Jugnot et al., 2015; Park Y. et al., 2020). Considering effectiveness, those studies with published results demonstrated the benefits of this methodology: Granel et al. and Guillaume-Jugnot et al. compared the evolution of the disease from the baseline to 12 months after treatment, reporting a significant improvement of 46.8% for the Scleroderma Health Assessment Questionnaire, of 51.3% for the Cochin Hand Function Scale (CHFS) score and of 63.2% for the Raynaud’s Condition score (Granel et al., 2015; Guillaume-Jugnot et al., 2015). Virzì et al. (2017) observed that co-injection of PRP and hAT-MSCs increased skin elasticity in perioral and malar areas (16.64% for the lip and the 17.80% for the cheek) (Virzì et al., 2017); and Park Y. et al. (2020) reported a slight improvement of Raynaud’s Condition Scale value but without significance (from 5 to 6), although health assessment questionnaire reported better values after 24 weeks (from 1.0 to 0.625) and better quality of life was observed.

In the case of hUCB-MSCs, 34 patients has been recruited and the number of cells for the treatment varied from 2 × 10^6^ cells/kg (2 doses) (Wehbe et al., 2016), one injection of 8 × 10^6^ cells/kg (Zhang et al., 2017) and one or two injections of 10^6^ cells/kg (NCT04356287). Adverse events noted were upper respiratory tract infections reported by five patients and diarrhea reported by one patient during follow-up visits (Zhang et al., 2017). Results of two patients treated by Wehbe et al. (2016) revealed that after one year of treatment, European Scleroderma Study Group activity index was reduced from 9.5 to 3.5 and 1.5, respectively (Wehbe et al., 2016) which correlates with the study of Zhang et al. (2017) where the mean Modified Rodnan skin score improved from 20.1 ± 3.1 to 13.8 ± 10.2 in 14 patients.

Finally, only one study evaluated the intramuscular injection of 50 × 10^6^ allogeneic hBM-MSCs for the treatment of 10 patients with systemic sclerosis (Van Rhijn-Brouwer et al., 2018) but there is not results posted (NCT03211793).

On balance, despite of the fact that there were few preclinical analysis, many clinical studies have evaluated the use of hMSCs as CT for the treatment of scleroderma, reporting promising results, however, increase the number of investigations and patients is required to provide reliable information about the potential benefits of these therapies.

### Hypertrophic Scars

Hypertrophic scars are abnormal wound responses in predisposed individuals. These fibrous growths result from a connective tissue response to trauma, inflammation, surgery, or burns and occasionally seem to occur spontaneously (English and Shenefelt, 1999).

#### Preclinical *in vivo* Studies

From 2015, many researches have explored the use of conditioned media derived from hMSCs culture, however, only two studies evaluated hMSCs as advanced therapy for the treatment of hypertrophic scars at preclinical level (Table 10; Domergue et al., 2016; Yates et al., 2017).

**TABLE 10:** Preclinical *in vivo* and clinical studies of hMSCs used as advanced therapy for hypertrophic scars in the last 5 years.

PRECLINICAL IN VIVO STUDIES										

Type of advanced therapy	Cells		Type of hMSC treatment evaluated			Animals	Disease models		Follow-up	References
Cell therapy	hAT-MSCs		1-hAT-MSCs group: subcutaneous injection of 10^6^ cells 2-Stromal vascular fraction (SVF) group subcutaneous injection of SVF containing 10^6^ hAT-MSCs 3-Control group: subcutaneous injection of phosphate buffered saline			30 Athymic Nude-*Foxn1*^*nu*^ (10 mice per group)	Human skin samples were grafted into mice for 7 weeks to develop hypertrophic scars		21 days	Domergue et al., 2016
	hBM-MSCs		1-hBM-MSCs group: addition to the wound of 10^6^ cells/ml 2-hBM-MSCs + collagen I-tenascin-C (TNC) group: addition to the wound of 10^6^ cells/ml + collagen I-tenascin-C (TNC) 3-hBM-MSCs + fibroblasts + hyaluronic acid (HA) group: addition to the wound of 10^6^ cells/ml + fibroblasts + HA 4-hBM-MSCs + fibroblasts + collagen I-TNC + hyaluronic acid (HA) group: addition to the wound of 10^6^ cells/ml + fibroblasts + collagen I-TNC + HA			– C57BL/6J wild-type and CXCR3 expression-abrogated mice	Wounds these mice presented a visible scar, a thickened epidermis, and a disorganized and hypercellular dermis		30 days	Yates et al., 2017

**CLINICAL STUDIES**										

**Type of advanced therapy**	**Cells**	**Type of clinical study**	**N (male/female)**	**Age (years)^a^**	**hMSC treatment**	**Safety (Treatment-related adverse events)**	**Indication**	**Effectiveness^a^**	**Follow-up^a^**	**References**

Cell therapy	Allogenic hUCB-MSCs	Phase II Randomized Clinical Trial (Parallel Assignment- Triple Blind)	90 (0/90)	28.04 ± 3.39	A transdermal application of 10^6^ cells for continuous 3 days (low-dose) or for continuous 6 days (high-dose) or placebo	No obvious side effects or adverse effects were reported	Caesarean Section Scars	Vancouver Scar Scale (VSS) score after 6 months was lower in the case of the high-dose group against low-dose and placebo groups (4.71, 5.18 and 6.43, respectively)	6 months and 36 months	Fan et al., 2018; Fan et al., 2020 NCT02772289 (Perinatal Tissue Mesenchyme Stem Cells in the Treatment for Caesarean Section Scars – Full Text View – ClinicalTrials.gov)^1^ NCT04034615 (Injection of Autologous Adipose-derived Stromal Vascular Fraction in the Finger of Systemic Sclerosis Patients – Full Text View – ClinicalTrials.gov)^2^

*^*a*^Expression of measures: mean ± standard deviation (range).*

*^1^Perinatal Tissue Mesenchyme Stem Cells in the Treatment for Caesarean Section Scars – Full Text View – ClinicalTrials.gov Available at: https://clinicaltrials.gov/ct2/show/NCT02772289 [Accessed February 14, 2021].*

*^2^Injection of Autologous Adipose-derived Stromal Vascular Fraction in the Finger of Systemic Sclerosis Patients – Full Text View – ClinicalTrials.gov Available at: https://clinicaltrials.gov/ct2/show/NCT03060551 [Accessed February 14, 2021].*

hAT-MSCs (Domergue et al., 2016) and hBM-MSCs (Yates et al., 2017) have been the cells investigated as CT for different *in vivo* models of hypertrophic scars (mean time of follow-up: 25.5 ± 6.3 days). In the first case, to develop hypertrophic scars, human skin grafts were engrafted into mice wounds for 7 weeks and compared the subcutaneous injection of 10^6^ hAT-MSCs against the injection of stromal vascular fraction (SVF) containing 10^6^ hAT-MSCs, reporting that one week after injection skin thickness tended to be lower in both groups but reduction of skin thickness was more significant in hAT-MSCs group, collagen fibers appeared less dense an organized and fibrotic scar was reduced more efficiently (Domergue et al., 2016).

Yates et al. (2017) used CXCR3 expression-abrogated mice where wounds generated presented a visible scar, with a thickened epidermis and a disorganized and hypercellular dermis. Researchers compared different treatments combining the injection of 10^6^ hBM-MSCs/ml with other cell types (fibroblasts), molecules (tenascin-C) or biomaterials (hyaluronic acid or collagen I hydrogel). Results indicated that collagen–tenascin-C matrix improved survival of MSCs and fibroblasts and the use of hBM-MSCs increased migratory and proliferative capacity of fibroblasts and also led to fewer inflammatory cells in the wound, preventing hyper-keratinization, as well as excessive matrix deposition.

On balance the use of hMSCs as cell therapy for the treatment of hypertrophic scars seems to be interesting, however, the lack of studies due to the minor impact into patient’s health compared to the rest of skin diseases analyzed, makes necessary to continue investigating.

#### Clinical Studies

As in the case of preclinical studies, the number of clinical studies in the last 5 years is limited (Table 10). Only one study, although design and results were published in two different articles (Fan et al., 2018, 2020) and clinical trials (NCT02772289, NCT04034615), has evaluated the transdermal injection of allogeneic hUCB-MSCs for the treatment of scars derived from caesarean sections at two different doses and compared the results with a placebo. 90 patients (30 for each group), were treated with an injection of 10^6^ cells for continuous 3 days (low-dose), for continuous 6 days (high-dose), or with placebo. Safety results revealed that no obvious side effects or adverse effects were reported and effectiveness was evaluated using the Vancouver Scar Scale (VSS) score, demonstrating that after 6 months values were lower (which means better) in the case of the high-dose group against low-dose and placebo groups (4.71, 5.18, and 6.43, respectively) (Fan et al., 2020).

Hypertrophic scars are not usually severe or life-threatening injuries and for this reason, number of studies evaluating the use of hMSC-based advanced therapies is limited. However, their esthetic implications could affect on mental health status of patients which has a direct impact on their quality of life, so the search of more successful treatments seems to be required.

### Other Dermatological Pathologies

Apart from the previous dermatological diseases reviewed there are others such as photoaging and acne which are less aggressive or conventional and effective treatments exist. In other cases, diseases such as vitiligo and hidradenitis suppurative (Cuenca-Barrales et al., 2020) affect to a minor population or there is not an effective treatment. In these cases, the number of studies reporting the use of hMSCs in the last 5 years is limited.

Among them, only one *in vitro* preclinical study reported the use of hMSCs (source was not indicated) for the treatment of vitiligo, a chronic disorder characterized by depigmented patches in the skin that are caused by deficiency or dysfunction in melanocytes. In this study, cocultured hMSCs with 10 different melanocyte pools reported an improved cell proliferation and suppressed apoptosis in melanocytes (Zhu et al., 2020).

In the case of clinical studies, only one administered autologous hAT-MSCs as CT for the treatment of photoaged skin. 20 patients (56 ± 2.53 years old) extensive exposed to sun and presenting Fitzpatrick class IV (*n* = 9) and class V (*n* = 11) skin, were treated. One year after subdermal injection of hAT-MSCs in preauricular region, non-adverse events were observed. Results revealed an extensive new production and regeneration of elaunin, oxytalan, and elastin fiber network located in the upper papillary dermis, concomitant with degradation of elastotic abnormal elastin deposits in the deeper dermal layers, which is the major characteristic of solar elastosis (Charles-De-Sá et al., 2020).

Other important cutaneous diseases not included in this review are alopecia and toxic skin injuries. In the first case, our group has recently published a comprehensive review where many advanced therapy has been analyzed including those using hMSCs (Martinez-Lopez et al., 2020). In the case of toxic skin conditions, many chemical agents could impair skin barrier and another review could be wrote about their possible treatment, however, in recent years, skin injuries generated by sulfur mustard (SM) agent have been analyzed (Rose et al., 2018) and the potential use of hMSCs-based advanced therapy could be interested because SM damage DNA of mesenchymal stem cells provoking less proliferative and migratory capacities (Schmidt et al., 2018; Schreier et al., 2018).

## Discussion

Conventional treatments for wounds, chronic ulcers or severe burns have been based on skin autografts, or allografts, however, the lack of donor’s tissue, the risk associated to the surgery and the possibility of immune rejection in the case of allografts (Brockmann et al., 2018) has made necessary to search for new alternatives. In other cases, such as psoriasis or atopic dermatitis, immunological profile plays a crucial role in the disease’s development, so the main commercialized drugs try to regulate this aspect, although there is not a 100% effective treatment.

Searching new alternatives for the treatment of skin disorders is an interesting field; the most promising results have been achieved for wounds, burns and ulcers where many tissue-engineered skin substitutes (TESSs) has been developed. Until now, most of these therapies are based on acellular biomaterials or different scaffolds in combination with autologous skin cells, fibroblasts and keratinocytes mainly (Germain et al., 2018; Meuli et al., 2019), to avoid immunological rejection. Although preclinical and clinical research has reported positive results, only few commercial TESSs are available on market due to the cost and the associated toxicity of biomaterials (Brockmann et al., 2018).

For these reasons, the use of hMSCs for the treatment of skin diseases and injuries is a promising strategy due to their regenerative and immunomodulatory capacities previously reviewed (Diehl et al., 2017). Since 2015, 46 preclinical *in vivo* studies (mouse and rat models) and 54 clinical studies (1316 patients) have evaluated the use of hMSCs as advanced therapy (Supplementary Table 1); however, wounds, ulcers, burns injuries, and psoriasis are the most analyzed pathological conditions.

Focusing only in clinical essays, cell therapy is the most investigated strategy (43 vs. 10 TE studies – Figure 1), probably due to the difficulties associated to the fabrication of TESS (TE) or the development of engineered cells (GT), which requires specific formation and manufacturing conditions. However, in the case of the wounds and ulcers, the number of studies is equated (8 CT vs. 8 TE) because of the small size of injuries, in comparison to severe burns, and the low immunogenic profile of hMSCs (Diehl et al., 2017).

Another important topic to consider is the autologous or allogeneic nature of cells reviewed: 40 studies have evaluated allogeneic hMSCs but only 12 have reported the use of autologous cells. In contrast to investigations where autologous adult cells are analyzed, where manufacturing time is longer, the immunomodulatory capacity of allogeneic hMSCs seems to be an advantage, because the rapid availability associated could increase the success rate. At this point, cryopreservation of allogeneic cells is an important issue to consider, because in some cases the potential benefits of these therapies have been affected (Weise et al., 2014). However, recent studies have determined that apart from cryopreservation, thawing is also more important to avoid a failure of these therapies, demonstrating that culturing these cells for 24 h after thawing, allowed to recover functional potency of hMSCs and, even, their immunosuppressive capacity was increased (Antebi et al., 2019).

Although CT based on the use of hMSCs is the predominant strategy for all pathologies reviewed, there is not a standardized number of cells, doses, or routes of administration, even if each disease is analyzed separately. This is a limitation, due to the impossibility of stablishing a common therapy and comparing results among studies; for example, in the case of burn injuries, treatment differs from injection around the wounds to topical application, meanwhile, in psoriasis, atopic dermatitis or scleroderma, although most of the studies evaluated intravenous administration; subcutaneous or intramuscular injections were also reported.

Considering intravenous administration as one of the main strategies, migration capacity of hMSCs must be studied. This property has been evaluated and increased for the treatment of strokes in a mouse model (Jablonska et al., 2018) or cerebral embolism in rats (Cui et al., 2017), demonstrating its benefits. In the case of cutaneous diseases, an *in vitro* study demonstrated that regenerative and migratory potential could be increased after culturing hMSCs with CCL5/Rantes (Kroeze et al., 2009). However, therapeutic potential of hMSCs in psoriasis or atopic dermatitis is focused in their capacity to counteract the recruitment capacity of the own pathological hMSCs which are responsible of provoking an excessive angiogenesis (Han et al., 2021) or inflammatory cell infiltration (Niu et al., 2019), and therefore, aggravate extent of the disease.

Analysis of hMSCs’ tissue sources revealed that mesenchymal stem cells isolated from adipose tissue (hAT-MSCs), bone marrow (hBM-MSCs) or umbilical cord blood (hUCB-MSCs) are the preferred sources, although in all cases results were positive. hAT-MSCs were studied in 30 cases (9 preclinical and 21 clinical), hBM-MSCs in 14 (4 vs. 10) and hUCB-MSCs in 32 (19 vs. 13) (Supplementary Table 1). This means that despite of the fact that for research, hUCB-MSCs are the most investigated because they are easy to obtain from tissue and cell banks; for clinical purposes, hAT-MSCs seems to be the best option.

An overview of each pathology reviewed and their clinical studies, revealed that for wounds and ulcers, allogeneic hAT-MSCs was the cell population most investigated and engraftment of TESSs constituted of hMSCs was the preferred strategy. In the case of burn injuries and psoriasis, allogeneic hAT-MSCs was also the most analyzed cell type but administered by topical application or intravenous injection, respectively. For scleroderma, autologous hAT-MSCs injected subcutaneously was the most investigate cell therapy, and for RDEB, allogeneic hBM-MSCs applied intravenously. Finally, for atopic dermatitis and hypertrophic scars there was not a preferred hMSC population, but the most analyzed administration routes were intravenous and transdermal injections, respectively.

Potential benefits of using hAT-MSCs are due to their ability of secreting a lot of factors which are involved in cell proliferation, migration, and improvement of cellular and microenvironment protection (Mazini et al., 2020), apart from their immunomodulatory properties to inactivate T cells (Jacobs et al., 2013) and modulate the inflammatory phenotype of local immune cells to a healing anti-inflammatory one (Mazini et al., 2020) which have implications in psoriasis, atopic dermatitis and scleroderma. The release of growth factors by hAT-MSCs increase the expression of genes related with skin regeneration, such as CD34, collagen type 1 or elastin and moreover, stimulate the proliferation of human fibroblasts (Choi et al., 2018) and the activation of AKT and ERK signaling pathways in human endothelial cells, keratinocytes, and fibroblasts which demonstrate their potential implications in wound healing (Ren et al., 2019). Most of these characteristics are also observed in other hMSCs, such as those derived from bone marrow (Pachón-Peña et al., 2011).

For all of this, apart from the direct use of hMSCs, many recent preclinical studies have evaluated the use of exosomes and conditioned mediums derived from them. These released molecules, alone or combine with other substances or scaffolds, have reported successful result in terms of wound closure and re-epithalization (Shabbir et al., 2015; Tao et al., 2017; Aryan et al., 2019; Dalirfardouei et al., 2019; Wang et al., 2019; Zhou et al., 2019; Jiang et al., 2020).

On balance, we consider that the use of hMSCs-based advanced therapies for the treatment of dermatological pathologies could increase patient’s health and quality of life in a significant way. On the one hand, the use of these cells helps to increase the regenerative capacity of the own patient’s hMSCs due to their role in wound healing and, on the other hand, the immunomodulatory capacities associated to them, are important to regulate the immunological response in diseases such as psoriasis or atopic dermatitis. However, despite of the fact that many promising advanced therapies based on the use of hMSCs have been developed and evaluated in the last years, this review remarks the necessity of increasing the investigation at preclinical level. The main animal models analyzed were mice and rats, however, to validate these therapies, pigs or apes would be better to resemble human wounds or diseases. In addition, more clinical research is required to evaluate safety and effectiveness, because in most of the cases, results have been not published yet or none control groups have been included. Particularly, for RDEB, the use of cells genetically modified in a clinical environment would be the most interesting therapy, however, the lack of information about the long-term effect of this strategy, makes difficult to develop clinical trials. In conclusion, filling all these aspects would help to determine the best strategy, source of hMSCs and doses of treatment for each cutaneous disease or injury.

## Author Contributions

ÁS-S had the conception, revised bibliography, and wrote the manuscript. TM-V revised bibliography and revised the different versions of the manuscript. MQ-V and MS-D revised the different versions of the manuscript. SA-S had the conception, revised bibliography, and the different versions of the manuscript. All authors contributed to the article and approved the submitted version.

## Conflict of Interest

The authors declare that the research was conducted in the absence of any commercial or financial relationships that could be construed as a potential conflict of interest.

## Supplementary Material

Supplementary Table 1 Summary table of the different types of hMSCs used as advanced therapy for the treatment of skin diseases and injuries and the number of studies reviewed from each population (2015–2020).

## Abbreviations

bFGF: basic fibroblast growth factor
CHFS: cochin Hand Function Scale
CT: cell therapy
DDEB: dominant dystrophic epidermolysis bullosa
DEB: dystrophic epidermolysis bullosa
DNCB 2: 4-dinitrochlorobenzene
EGF: epidermal growth factor
GT: gene therapy
hA-MSCs: human amnion mesenchymal stem cells
hAT-MSCs: human adipose mesenchymal stem cells
hBM-MSCs: human bone marrow mesenchymal stem cells
hDP-MSCs: human dermal papilla mesenchymal stem cells
hDT-MSCs: human deciduous teeth mesenchymal stem cells
hG-MSCs: human gingival mucosa mesenchymal stem cells
hJM-MSCs: human jaw bone marrow mesenchymal stem cells
hMen-MSCs: human menstrual fluid mesenchymal stem cells
hMSCs: human mesenchymal stem cells
hP-MSCs: human placenta mesenchymal stem cells
hPT-MSCs: human palatine tonsil derived mesenchymal stem cells
hUCB-MSCs: human umbilical cord blood mesenchymal stem cells
hWJ-MSCs: human wharton’s jelly mesenchymal stem cells
IMQ: imiquimod
KLCs: keratinocyte-like cells
MCs: mast cells
MRSC: modified rodnan skin score
PASI: psoriasis area and severity index
PDLLA, poly-D: L-lactic acid
PRP: platelet-rich plasma
RDEB: recessive dystrophic epidermolysis bullosa
SCORAD: severity scoring for atopic dermatitis
SDS: sodium dodecyl sulfate
SHAQ: scleroderma health assessment questionnaire
SM: sulfur mustard
SOD3: superoxide dismutase
SSc: systemic sclerosis
TBSA: total body surface area
TE: tissue engineering
TESSs: tissue-engineered skin substitutes
TGF- β: transforming growth factor beta
VEGF: vascular endothelial growth factor
VSS: vancouver scar scale.
