# Supplementary material for: Current Advanced Therapies Based on Human Mesenchymal Stem Cells for Skin Diseases
Source: Front Cell Dev Biol. 2021 Mar 9;9:643125. doi: 10.3389/fcell.2021.643125 (PMC7985058; doi:10.3389/fcell.2021.643125)
Supplement: Supplementary Table 1 — Summary table of the different types of hMSCs used as advanced therapy for the treatment of skin diseases and injuries and the number of studies reviewed from each population (2015–2020). [file Table_1.DOCX]

Supplementary Material

# Supplementary Figures and Tables

## Table S1. Summary table of the different types of hMSCs used as advanced therapy for the treatment of skin diseases and injuries and the number of studies reviewed from each population (2015-2020).

| **Type of hMSC** | **Dermatological Pathology** | **Type of advanced therapy** | **Preclinical *in vivo* studies**  **(2015-2020)** | **Clinical studies**  **(2015-2020)** | | | **Patient’s Recruitment** |
| --- | --- | --- | --- | --- | --- | --- | --- |
|  |  |  |  | **Autologous** | **Allogeneic** | **Not Indicated** |  |
| **hA-MSCs** | **Wounds and ulcers** | **Cell Therapy** |  |  |  |  | **-** |
|  |  | **Tissue Engineering** | **1** |  |  |  |  |
|  |  | **Gene Therapy** |  |  |  |  |  |
|  | **Psoriasis** | **Cell Therapy** | **1** |  |  |  | **-** |
|  |  | **Tissue Engineering** |  |  |  |  |  |
|  |  | **Gene Therapy** |  |  |  |  |  |
| **hAT-MSCs** | **Wounds and ulcers** | **Cell Therapy** | **3** |  |  |  | **425** |
|  |  | **Tissue Engineering** | **1** | **1** | **6** |  |  |
|  |  | **Gene Therapy** |  |  |  |  |  |
|  | **Burns** | **Cell Therapy** |  |  |  | **1** | **43** |
|  |  | **Tissue Engineering** | **1** |  | **2** |  |  |
|  |  | **Gene Therapy** |  |  |  |  |  |
|  | **Psoriasis** | **Cell Therapy** |  | **2** | **3** |  | **23** |
|  |  | **Tissue Engineering** |  |  |  |  |  |
|  |  | **Gene Therapy** |  |  |  |  |  |
|  | **Atopic Dermatitis** | **Cell Therapy** | **2** |  |  | **1** | **118** |
|  |  | **Tissue Engineering** |  |  |  |  |  |
|  |  | **Gene Therapy** |  |  |  |  |  |
|  | **Scleroderma** | **Cell Therapy** | **1** | **5** |  |  | **44** |
|  |  | **Tissue Engineering** |  |  |  |  |  |
|  |  | **Gene Therapy** |  |  |  |  |  |
|  | **Hypertrophic scars** | **Cell Therapy** | **1** |  |  |  | **-** |
|  |  | **Tissue Engineering** |  |  |  |  |  |
|  |  | **Gene Therapy** |  |  |  |  |  |
| **hBM-MSCs** | **Wounds and ulcers** | **Cell Therapy** | **1** |  | **1** |  | **1** |
|  |  | **Tissue Engineering** |  |  |  |  |  |
|  |  | **Gene Therapy** |  |  |  |  |  |
|  | **Burns** | **Cell Therapy** |  | **1** | **1** |  | **21** |
|  |  | **Tissue Engineering** |  |  |  |  |  |
|  |  | **Gene Therapy** |  |  |  |  |  |
|  | **RDEB** | **Cell Therapy** | **1** |  | **5** |  | **46** |
|  |  | **Tissue Engineering** |  |  |  |  |  |
|  |  | **Gene Therapy** |  |  |  |  |  |
|  | **Atopic Dermatitis** | **Cell Therapy** |  |  | **1** |  | **92** |
|  |  | **Tissue Engineering** |  |  |  |  |  |
|  |  | **Gene Therapy** |  |  |  |  |  |
|  | **Scleroderma** | **Cell Therapy** | **1** |  | **1** |  | **10** |
|  |  | **Tissue Engineering** |  |  |  |  |  |
|  |  | **Gene Therapy** |  |  |  |  |  |
|  | **Hypertrophic scars** | **Cell Therapy** | **1** |  |  |  | **-** |
|  |  | **Tissue Engineering** |  |  |  |  |  |
|  |  | **Gene Therapy** |  |  |  |  |  |
| **hDP-MSCs** | **Wounds and ulcers** | **Cell Therapy** |  |  |  |  | **-** |
|  |  | **Tissue Engineering** | **1** |  |  |  |  |
|  |  | **Gene Therapy** |  |  |  |  |  |
| **hDT-MSCs** | **Burns** | **Cell Therapy** |  |  |  |  | **-** |
|  |  | **Tissue Engineering** | **1** |  |  |  |  |
|  |  | **Gene Therapy** |  |  |  |  |  |
| **hG-MSCs** | **Psoriasis** | **Cell Therapy** |  |  | **1** |  | **1** |
|  |  | **Tissue Engineering** |  |  |  |  |  |
|  |  | **Gene Therapy** |  |  |  |  |  |
| **hJM-MSCs** | **Wounds and ulcers** | **Cell Therapy** | **1** |  |  |  | **-** |
|  |  | **Tissue Engineering** |  |  |  |  |  |
|  |  | **Gene Therapy** |  |  |  |  |  |
| **hMen-MSCs** | **Wounds and ulcers** | **Cell Therapy** | **1** |  |  |  | **-** |
|  |  | **Tissue Engineering** |  |  |  |  |  |
|  |  | **Gene Therapy** |  |  |  |  |  |
| **hP-MSCs** | **Wounds and ulcers** | **Cell Therapy** |  |  | **1** |  | **43** |
|  |  | **Tissue Engineering** | **1** |  |  |  |  |
|  |  | **Gene Therapy** |  |  |  |  |  |
| **hPT-MSCs** | **Psoriasis** | **Cell Therapy** | **1** |  |  |  | **-** |
|  |  | **Tissue Engineering** |  |  |  |  |  |
|  |  | **Gene Therapy** |  |  |  |  |  |
| **hUCB‐MSCs** | **Wounds and ulcers** | **Cell Therapy** | **3** |  | **2** |  | **130** |
|  |  | **Tissue Engineering** | **2** |  |  |  |  |
|  |  | **Gene Therapy** |  |  |  |  |  |
|  | **Burns** | **Cell Therapy** |  |  | **2** |  | **21** |
|  |  | **Tissue Engineering** | **1** |  |  |  |  |
|  |  | **Gene Therapy** |  |  |  |  |  |
|  | **RDEB** | **Cell Therapy** | **1** |  | **1** |  | **5** |
|  |  | **Tissue Engineering** | **1** |  |  |  |  |
|  |  | **Gene Therapy** | **1** |  |  |  |  |
|  | **Psoriasis** | **Cell Therapy** | **3** |  | **3** |  | **23** |
|  |  | **Tissue Engineering** |  |  |  |  |  |
|  |  | **Gene Therapy** | **1** |  |  |  |  |
|  | **Atopic Dermatitis** | **Cell Therapy** | **4** |  | **1** |  | **33** |
|  |  | **Tissue Engineering** |  |  |  |  |  |
|  |  | **Gene Therapy** | **2** |  |  |  |  |
|  | **Scleroderma** | **Cell Therapy** |  |  | **3** |  | **34** |
|  |  | **Tissue Engineering** |  |  |  |  |  |
|  |  | **Gene Therapy** |  |  |  |  |  |
|  | **Hypertrophic Scars** | **Cell Therapy** |  |  | **1** |  | **90** |
|  |  | **Tissue Engineering** |  |  |  |  |  |
|  |  | **Gene Therapy** |  |  |  |  |  |
| **hWJ-MSCs** | **Wounds and ulcers** | **Cell Therapy** | **1** |  | **1** |  | **6** |
|  |  | **Tissue Engineering** | **1** |  | **1** |  |  |
|  |  | **Gene Therapy** |  |  |  |  |  |
|  | **Burns** | **Cell Therapy** | **1** |  |  |  | **-** |
|  |  | **Tissue Engineering** | **1** |  |  |  |  |
|  |  | **Gene Therapy** |  |  |  |  |  |
|  | **Atopic Dermatitis** | **Cell Therapy** | **1** |  |  |  | **-** |
|  |  | **Tissue Engineering** |  |  |  |  |  |
|  |  | **Gene Therapy** |  |  |  |  |  |
| **hMSCs**  **(not defined)** | **Wounds and ulcers** | **Cell Therapy** |  | **1** | **2** |  | **67** |
|  |  | **Tissue Engineering** | **1** |  |  |  |  |
|  |  | **Gene Therapy** |  |  |  |  |  |
|  | **RDEB** | **Cell Therapy** |  |  | **1** |  | **16** |
|  |  | **Tissue Engineering** |  |  |  |  |  |
|  |  | **Gene Therapy** |  |  |  |  |  |
|  | **Atopic Dermatitis** | **Cell Therapy** |  | **2** |  |  | **24** |
|  |  | **Tissue Engineering** |  |  |  |  |  |
|  |  | **Gene Therapy** |  |  |  |  |  |
| **TOTAL** | | | **46** | **12** | **40** | **2** | **1316** |
